# Rotundic acid improves nonalcoholic steatohepatitis in mice by regulating glycolysis and the TLR4/AP1 signaling pathway

**DOI:** 10.1186/s12944-023-01976-z

**Published:** 2023-12-04

**Authors:** Xing-Yang Shi, Xiao-Min Zheng, Hui-Jie Liu, Xue Han, Lei Zhang, Bei Hu, Shan Li

**Affiliations:** 1 MOE International Joint Laboratory for Synthetic Biology and Medicines, School of Biology and Biological Engineering, South China University of Technology, Guangzhou, 510006 P. R. China; 2 Department of Emergency Medicine, Guangdong Provincial People’s Hospital (Guangdong Academy of Medical Sciences), Southern Medical University, Guangzhou, 510030 PR China; 3 School of Medicine, South China University of Technology, Guangzhou, 510006 PR China; 4 NMPA Key Laboratory for Quality Control of Blood Products, Guangdong Institute for Drug Control, Guangzhou, 510663 PR China

**Keywords:** NASH, RA, Glycolysis, TLR4/AP1, Proteomics, Transcriptomics

## Abstract

**Background:**

Steatosis and inflammation are the hallmarks of nonalcoholic steatohepatitis (NASH). Rotundic acid (RA) is among the key triterpenes of Ilicis Rotundae Cortex and has exhibited multipronged effects in terms of lowering the lipid content and alleviating inflammation. The study objective is to systematically evaluate the potential mechanisms through which RA affects the development and progression of NASH.

**Methods:**

Transcriptomic and proteomic analyses of primary hepatocytes isolated from the control, high-fat diet-induced NASH, and RA treatment groups were performed through Gene Ontology analysis and pathway enrichment. Hub genes were identified through network analysis. Integrative analysis revealed key RA-regulated pathways, which were verified by gene and protein expression studies and cell assays.

**Results:**

Hub genes were identified and enriched in the Toll-like receptor 4 (TLR4)/activator protein-1 (AP1) signaling pathway and glycolysis pathway. RA reversed glycolysis and attenuated the TLR4/AP1 pathway, thereby reducing lipid accumulation and inflammation. Additionally, lactate release in L-02 cells increased with NaAsO_2_-treated and significantly decreased with RA treatment, thus revealing that RA had a major impact on glycolysis.

**Conclusions:**

RA is effective in lowering the lipid content and reducing inflammation in mice with NASH by ameliorating glycolysis and TLR4/AP1 pathways, which contributes to the existing knowledge and potentially sheds light on the development of therapeutic interventions for patients with NASH.

**Supplementary Information:**

The online version contains supplementary material available at 10.1186/s12944-023-01976-z.

## Introduction

Nonalcoholic fatty liver disease (NAFLD) is a growing worldwide health concern and a significant trigger for liver and cardiometabolic diseases [1]. Nonalcoholic steatohepatitis (NASH) is among the more aggressive forms of disease progression in NAFLD. This condition is distinguished by steatosis, inflammation, and cellular damage and may further lead to hepatic fibrosis, cirrhosis, and hepatocellular carcinoma [2, 3]. Currently, US FDA-approved drugs or biological treatments are lacking [4, 5]. To date, obeticholic acid, a farnesoid X receptor agonist, is the only drug granted breakthrough status by the US FDA. However, this drug can cause dyslipidaemia, and its launch has been delayed. Therefore, research to identify new drugs for NASH is urgently needed.

The pathogenesis of NASH involves a complex interaction among systemic metabolic disorders (e.g., obesity), environmental factors (e.g., diet) [6, 7] and predisposing genetic variants (e.g., transmembrane 6 superfamily member 2) [8, 9], resulting in disturbed glucose and lipid homeostasis and an inordinate accumulation of lipids in hepatocytes. Among these interacting components, the glycolytic pathway is a key regulatory point [10]. Glycolysis is markedly enhanced in hepatocytes in fatty liver, being the cause of strengthened de novo lipogenesis and increased liver inflammation. NASH is an inflammatory process involving the activation of inflammatory signaling pathways and immune cell recruitment. Recent research has revealed a connection between fatty acid metabolism and immunity, specifically through Toll-like receptor 4 (TLR4) [11].

llicis Rotundae Cortex (IRC), a traditional Chinese medicine, is the dehydrated bark of *Ilex rotunda* Thunb. (I. rotunda). This medicine is widely used to treat cardiovascular diseases and hepatitis [12]. Triterpenoids are considered active ingredients of IRC [13]. Among triterpenoids, rotundic acid (RA) is one of the key triterpenes that found in abundance in IRC [14, 15]. RA has exhibited multipronged effects in terms of hepatoprotection, improving lipid metabolism, and alleviating inflammation [16–18]. Early research has revealed that RA decreases the triglyceride and total cholesterol contents in the NASH model and reduces the protein and gene expression levels of inflammatory factors [16]. However, only the effect of RA on lipid metabolism has been explored. Hence, a more detailed mechanism of the effect of RA on NASH needs to be explored systematically by in vivo studies.

The study elucidated the potential mechanisms by which RA affects the development of NASH, investigated the effects of RA on glycolysis and the TLR4/activator protein 1 (AP1) pathway in primary liver cells and provided a development for therapeutic interventions for NASH.

## Materials and methods

### Chemicals and reagents

RA was supplied by the preliminary laboratory (purity: 98.7%) (refer to Supplementary File 1 and Supplementary Figs. 1 and 2 for details) [19]. Anti-cyclophilin B (CYPB) (DF12151), anti-phospho-c-Fos (AF3053), anti-hexokinase 2 (HK2) (DF6176), anti-c-Jun (AF6090), anti-pyruvate kinase 2 (PKM2) (DF6071), and anti-phospho-c-Jun (AF3095) antibodies were provided by Affinity Co. (Nanjing, Jiangsu, China). Anti-myeloid differentiation primary response gene 88 (MyD88) (A19082), anti-phosphofructokinase (PFKL) (A7708), anti-carbohydrate-responsive element-binding protein (ChREBP) (A7630), anti-Toll-like receptor 4 (TLR4) (A17436), and anti-c-Fos were provided by ABclonal Technology Co. (Wuhan, Hubei, China). 1 × TBST buffer was used to dilute every antibody used in the investigation.

### Isolation of primary liver cells

**Fig. 1:**
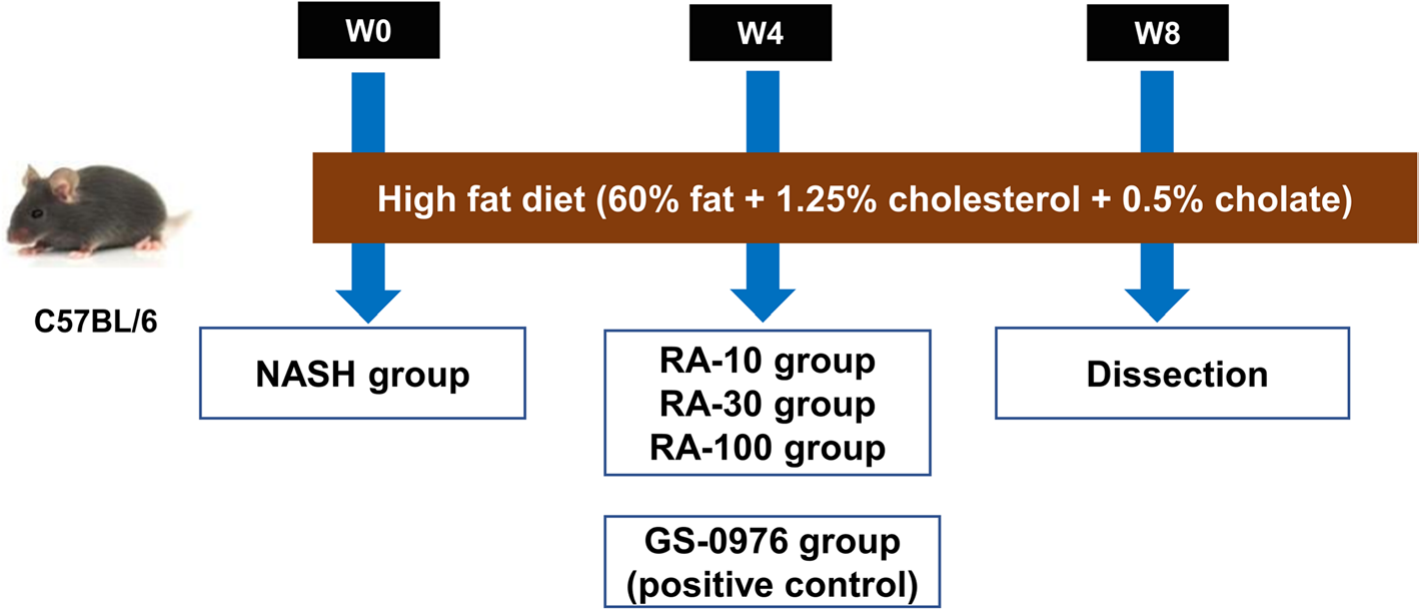
A schematic diagram of the animal experiments

Animal experiments were conducted in the early research [16] (refer to Fig. 1 and Supplementary File 1 for details).

At the end of these experiments, the animals were anesthetized with pentobarbital sodium solution (5 mg/100 g), and primary liver cells were collected. Two steps of collagenase perfusion were performed [20–22]. Hanks Balanced Salt Solution was first used to perfuse the liver until no blood was flushed out of the liver. Then, EGTA was poured at a 15 mL/min rate. For digestion of the liver tissues, collagenase medium containing DMEM, 1 g/L glucose, and 100 units/mL collagenase IV was perfused into the liver. The liver was gently scraped with a scalpel to release hepatocytes into the medium. A 75-µm pore size filter was used to filter the hepatocyte homogenate, and gradient centrifugation was performed to separate viable hepatocytes.

The culture medium was used to wash the hepatocytes, Kupffer cells, and hepatic stellate cells. The trypan blue (0.04% in incubation buffer) exclusion method was employed to assess cell viability. Primary hepatocytes were used for further proteomics and transcriptomics analyses.

### Transcriptomics

Total RNA from the primary hepatocytes was acquired by using the RNAprep Pure Cell/Bacteria Kit (TIANGEN Biotech, Beijing, China). Conducting and sequencing of a complementary DNA (cDNA) library was carried out by BGI-Shenzhen (Shenzhen, Guangdong, China). Raw data were filtered using the software SOAPnuke independently developed by BGI-Shenzhen (Shenzhen, Guangdong, China). For aligning clean reads to the gene set and reference genome, the sequence alignment software HISAT was used, such that gene or transcript expression in the sample can be quantified. Significantly differentially expressed genes (DEGs) were those with a false discovery rate (FDR) of ≤ 0.001 and a fold change of ≥ 1.5.

### Proteomics

Primary hepatocytes were dissolved in a 1 × cocktail (containing 1% SDS, 2 mM EDTA, and 10 mM DTT). All samples were quantified, reduced, alkylated, trypsin-digested, and desalted.

The desalted samples were subjected to high pH RP separation by using the Shimadzu LC-20AB HPLC system and analyzed via a Q Exactive HF mass spectrometer with an Ultimate 3000 RSLCnano system (Thermo Fisher Scientific, San Jose, CA, USA). Data-dependent acquisition (DDA) and data-independent acquisition (DIA) were carried out by BGI-Shenzhen (Shenzhen, Guangdong, China).

A spectral library was constructed by identifying DDA data in MaxQuant (version 1.5.3.30) via the Andromeda search engine and then analyzing these identified data in Spectronaut [23]. A spectral library of identified peptides satisfying FDR ≤ 1% was constructed. The DIA data were quantitatively analyzed using Spectronaut to obtain significant results with FDR ≤ 1% [24]. The process involved data preprocessing by MSstats dependent on the predefined comparison group, and then, the significance test was conducted on the basis of the model [25]. Subsequently, significantly differentially expressed proteins (DEPs) were defined as those with a fold change ≥ 1.5.

### Bioinformatics analysis

All candidate genes were classified into Gene Ontology (GO) database entries, and the gene counts per entry were calculated. After the candidate genes were compared to the background genes of *Mus musculus*, a hypergeometric test was conducted to find significant enrichment of GO functions. The *P* value was calculated by employing the basis function phyper of R, which was then corrected by multiple testing. The corrected package is known as the Q value. Finally, a Q value of ≤ 0.05 was used as the cut-off value. GO terms that satisfied this parameter were considered to be markedly enriched in candidate genes.

Pathway enrichment analysis was carried out using the same aforementioned method by utilizing the Kyoto Encyclopedia of Genes and Genomes (KEGG) pathway database.

Following the KEGG enrichment analysis, the protein − protein interaction (PPI) network was constructed using STRING [26]. As a criterion of statistical significance, the interaction score with the highest confidence (0.900) was considered the minimum requirement. The PPI network was imported into Cytoscape (version 3.9.0), and hub genes were explored by the CytoHubba plugin app. DEGs and DEPs identified through the aforementioned method were mapped to the KEGG pathway by using the Pathview website [27].

### Quantitative real-time polymerase chain reaction (qPCR) analysis

The total RNA of primary liver cells was utilized to synthesize cDNA using TIANScript II RT Kit (TIANGEN Biotech, Beijing, China). For qPCR, TB Green Fast qPCR Mix (TaKaRa, Tokyo, Japan) was used and was performed on a LightCycler 96 System (Roche Diagnostics, Mannheim, BW, Germany) and a Applied Biosystems 7300 Real-Time PCR System (Thermo Fisher Scientific, Waltham, MA, USA). CYPB was considered the reference gene, and target genes were determined using the 2^−△△Ct^ method. Table 1 enlists the primer sequences.

**Table 1:** Sequences of the primers

Gene (*Mus musculus*)	Primers	Sequences (5' to 3')
HK2	Forward	GAGAAAGCTCAGCATCGTGG
	Reverse	TCCATTTGTACTCCGTGGCT
PFKFB3	Forward	CGAGATCGATGCTGGTGTGT
	Reverse	CTCCAGGCGTTGGACAAGAT
PKM2	Forward	GGCTCCTATCATTGCCGTGA
	Reverse	AAGGTACAGGCACTACACGC
PFKL	Forward	CGCTGCAATGGAGAGTTGTG
	Reverse	CCTCAAAGACGTAGGCAGCA
ENO1	Forward	CGCGTCTGTCCTTAAGGCTCTC
	Reverse	GCGGTGTACAGATCGACCTCA
LDHA	Forward	GGACAGTGCCTACGAGGTGAT
	Reverse	GGATGCACCCGCCTAAGG
ChREBP	Forward	TGCCATCAACTTGTGCCAGC
	Reverse	TGCGGTAGACACCATCCCAT
MLX	Forward	CACAAGGAGAAGAAAAAGCAGGAG
	Reverse	AATCTCTCGTAGAGTCTGTGGC
TLR4	Forward	TCTGGGGAGGCACATCTTCT
	Reverse	AGGTCCAAGTTGCCGTTTCT
MyD88	Forward	AACGCCGGAACTTTTCGATG
	Reverse	TTCTGTTGGACACCTGGAGA
c-JUN	Forward	AAAACCTTGAAAGCGCAAAA
	Reverse	CGCAACCAGTCAAGTTCTCA
c-FOS	Forward	GGGGACAGCCTTTCCTACTA
	Reverse	TGGGGATAAAGTTGGCACTA
CYPB	Forward	TTCTTCATAACCACAGTCAAGACC
	Reverse	ACCTTCCGTACCACATCCAT

### Western blotting

Proteins were separated on a 12% SDS-PAGE gel and detected on polyvinylidene difluoride membranes (Bio-Rad, Hercules, CA, USA). First, non-fat dry milk/TBST buffer (3%) was utilized to block the membranes, then primary antibodies were kept with the membrane at 4 °C with gentle shaking overnight, followed by 1 h treatment with corresponding secondary antibodies at ambient temperature. Bands were visualized using chemiluminescent western blot reagents (Thermo Fisher, Waltham, MA, USA), and the images were recorded with a chemiluminescence system. Protein bands were quantified via the ImageJ software (Rawak Software, Inc., Stuttgart, Germany).

### Cell Counting Kit-8 (CCK-8) assays

Human normal hepatocytes (L-02) were purchased from Shanghai Gaining Biotechnology (China) and cultivated in RPMI-1640 medium (Gibco, Waltham, MA, USA) augmented with foetal bovine serum (10%), streptomycin (100 μg/mL), and penicillin (100 U/mL) at 37 °C, and in 5% CO_2_. L-02 cells (8000 cells/well) were cultivated in the 96-well plates. Once the cells completely adhered to the plates, different doses of NaAsO_2_ were introduced to the plates for 24 h. Lastly, CCK-8 buffer was added to final concentrations of 10% (v/v) and the absorbance was taken via a microplate reader at 450 nm.

### Extracellular lactate production assays

A Lactate Production Kit (Njjcbio, Nanjing, China) was used to measure the amount of lactate released into the culture medium. The absorbance was read at 530 nm by using an ultraviolet‒visible spectrophotometer.

### Statistical analysis

The acquired data were depicted as the mean ± SEM. All the statistical assessments were conducted via the GraphPad Prism 9 (GraphPad Software, Inc., San Diego, CA, USA). For inter-group differences, a two-tailed Student's t-test was applied. Statistically significant values were set as *P* < 0.05.

## Results

### Effects of RA on NASH at the transcriptome level

According to the early study, the RA-30 group (30 mg/kg RA) showed alleviation of NASH signs, which was similar to that exhibited by the positive control GS-0976 group [16]. Therefore, primary hepatocytes isolated from the control group, HFD-induced NASH group, and RA-30 (30 mg/kg RA) group were used for transcriptomics and proteomics analyses.

The yields of primary hepatocytes in the control, NASH, and RA groups were approximately 4.5 × 10^7^, 1 × 10^7^, and 1.44 × 10^7^ cells/per mouse, respectively (Supplementary Table 1). The quantity was sufficiently high for conducting downstream transcriptome or proteome analysis.

On average, 11.75 Gb of sequenced nucleotides was obtained from the primary hepatocytes of each sample, with a genome mapping rate of 97.66% (Supplementary Table 2).

**Fig. 2:**
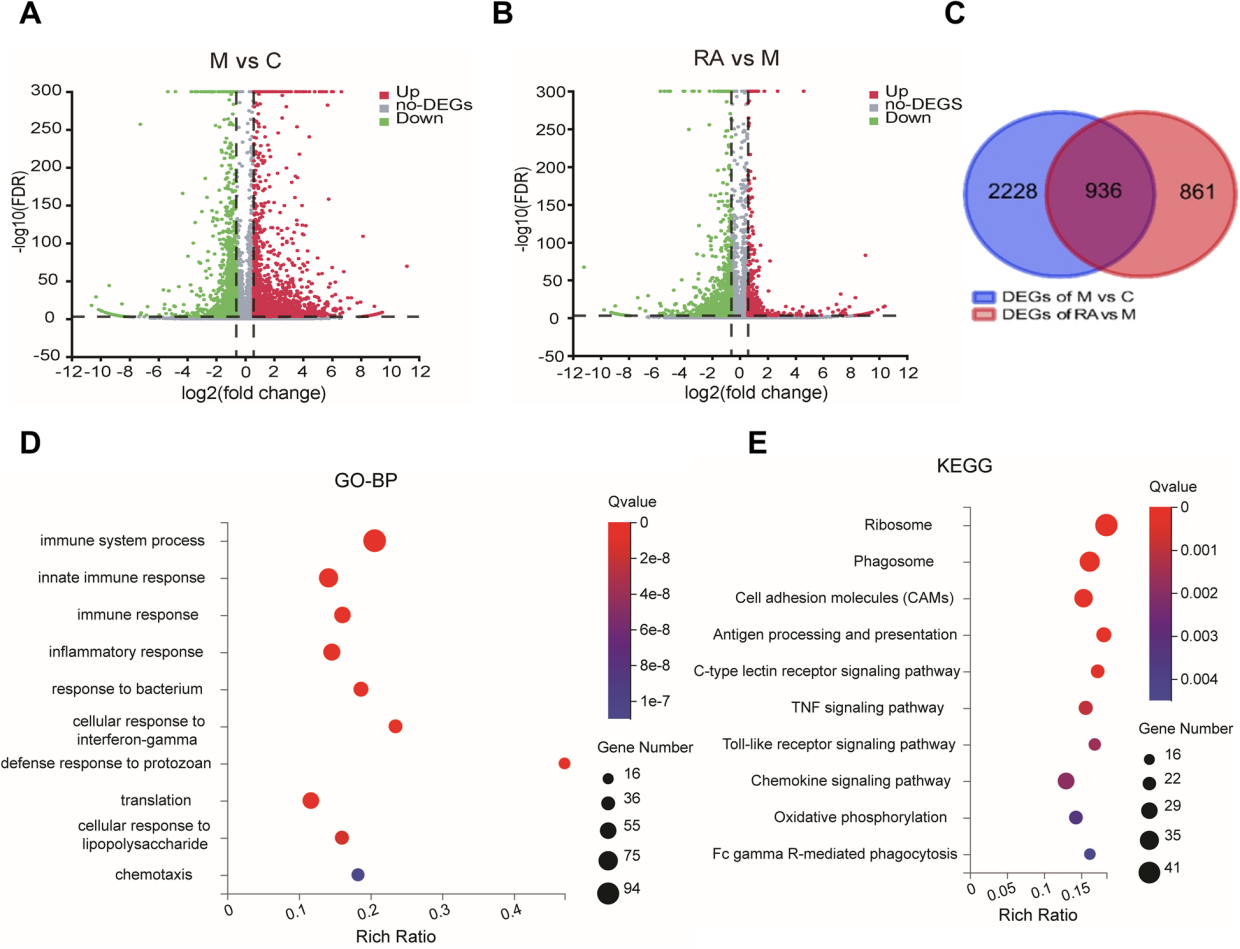
An overview of transcriptome and functional enrichment analysis of DEGs. **A-B** Global gene expression changes of M *vs.* C (**A**) and RA *vs.* M (**B**) are plotted as volcano plots. DEGs in red are upregulated, DEGs in green are downregulated, and non-DEGs are highlighted in gray. **C** Venn diagram of intersecting DEGs of M *vs.* C and RA *vs.* M. **D-E** Top 10 categories for GO biological processes (**D**) and KEGG pathways (**E**) of DEGs depicted by bubble diagrams. The color indicates the Q value, and the size indicates the gene number of each pathway. C: Control group. M: NASH group. RA: RA-30 group. *N* = 10 per group

Of these, 3164 genes showed significantly changes between the control and NASH groups (1678 and 1486 were upregulated and downregulated, respectively) (Fig. 2A). A significant difference in 1797 genes was observed between the NASH and RA groups (340 and 1457 genes were upregulated and downregulated, respectively) (Fig. 2B). Overall, 936 intersecting DEGs were probably target genes involved in NASH pathogenesis and RA treatment (Fig. 2C and Supplementary Table 3).

For functional classifications, GO analysis was performed for the 936 intersecting DEGs. The top 10 GO terms were focused on, which mainly included the immune process and the inflammation response (Fig. 2D). The physiological roles of these DEGs were identified based on the KEGG analysis. (Fig. 2E). Among the top 10 pathways, 6 pathways (namely, Fc gamma R-mediated phagocytosis, phagosome, antigen processing and presentation, and chemokine, Toll-like receptor, and C-type lectin receptor signaling pathways) were related to the immune system. Moreover, the tumor necrosis factor (TNF) signaling pathway can trigger multiple intracellular signals affecting inflammation and immunity. These results are consistent with those of the GO analysis.

According to the comprehensive GO and KEGG analyses, RA might exert anti-inflammatory effects by regulating the inflammation-inducing immune response.

### Network diagram analysis of the transcriptome

**Fig. 3:**
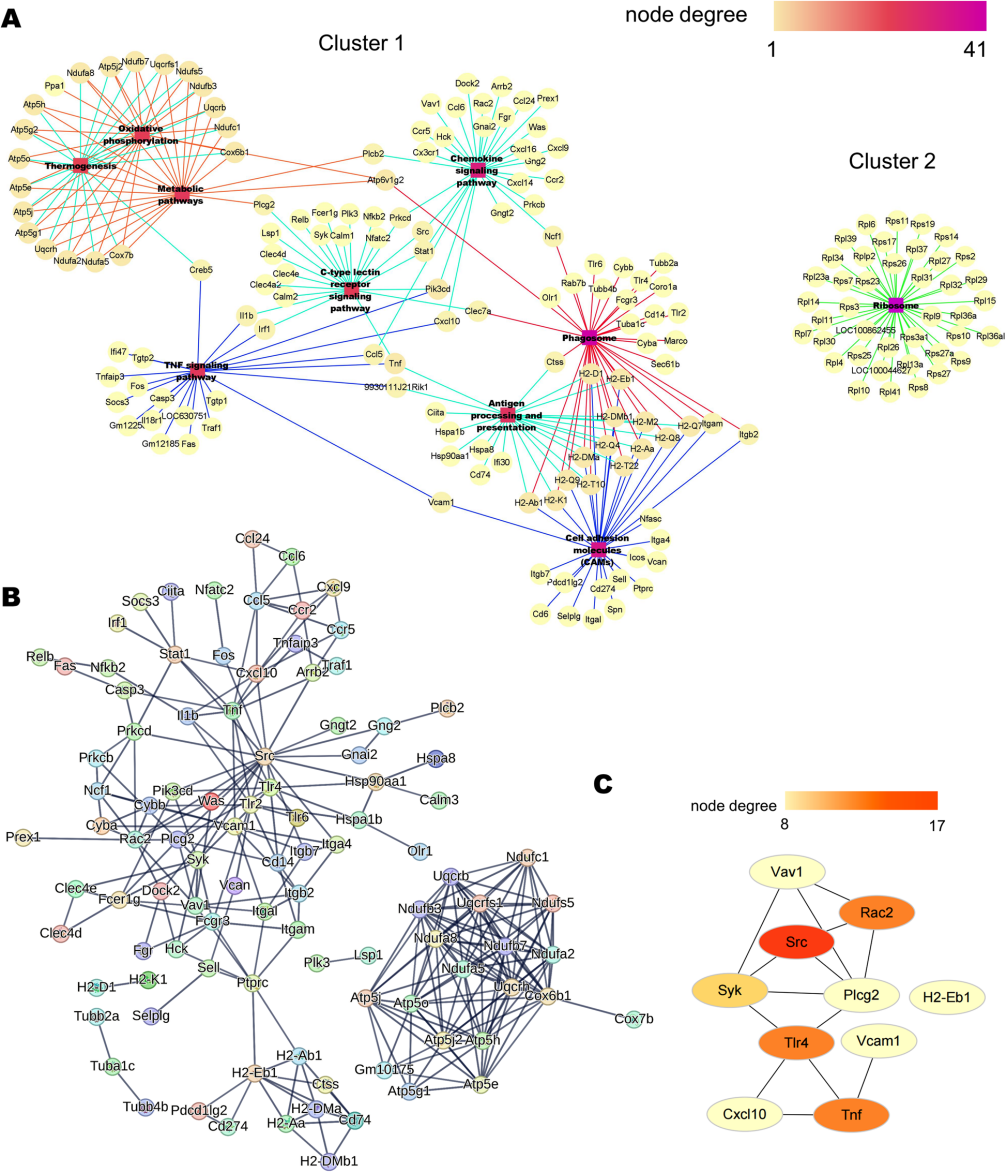
Network analysis of transcriptomics. **A** Pathway relation network of the top 10 KEGG pathways in DEGs. **B** The PPI network for DEGs in Cluster 1. **C** PPI hub genes ranked by degree in Cytoscape (ver.3.9.0). Node degree is represented by the redness of the nodes

The top 10 pathways with the most genes were categorized into two clusters through KEGG enrichment analysis (Fig. 3A).

Cluster 1 primarily comprised the immune system (antigen processing and presentation, C-type lectin receptor, and chemokine signaling pathway), metabolic pathway (oxidative phosphorylation), and TNF signaling pathway related to inflammation and immunity. Cluster 2 included the ribosome, which may contribute to NASH development, but the mechanism of action of the ribosome in NASH has still remained unclear [28–30].

Both KEGG and GO analyses indicated that the regulation of the immune/inflammatory response was largely enriched. Therefore, Cluster 1 was selected as the core cluster for identifying hub genes. The STRING tool was utilized for establishing the PPI network. This network consisted of 125 nodes interacting with each other through 271 edges (Fig. 3B). The DEGs were enriched in oxidative phosphorylation and formed an independent cluster. Oxidative phosphorylation is associated with oxidative stress-induced liver injury [31]. However, RA had no obvious effect on hepatic injury and could not reverse the decrease in oxidative phosphorylation in the NASH group (Supplementary Fig. 3) [16]. Therefore, the other cluster containing more genes was selected and imported into Cytoscape. The top 10 hub genes were identified based on the Degree in cytoHubba: Rous sarcoma oncogene (SRC), TLR4, Rac family small GTPase 2 (RAC2), TNF, spleen tyrosine kinase (SYK), phospholipase C gamma 2 (PLCG2), histocompatibility 2 (H2-Eb1), vascular cell adhesion molecule 1 (VCAM1), vav 1 oncogene (VAV1), and C-X-C motif chemokine ligand 10 (CXCL10) (Fig. 3C and Supplementary Table 4). Among these hub genes, TLR4 represents the intersection of metabolism and immunity, thereby playing a vital role in HFD-induced inflammation. TLR4 also regulates the expression of the inflammatory cytokine TNFα through the classical pathway [11, 32, 33]. The PLCG2-IP3-Ca^2+^ cascade activates TLR4 translocation, and TLR4 mediates the expression of IRF3 regulatory genes with SYK [34–36]. In addition, CXCL10 plays a crucial role in recruiting macrophages and is associated with the induction of proinflammatory cytokines (TNFα, IL-1β) [37].

Hence, hub genes were enriched in TLR4-mediated inflammation, a process that generally contributes to fatty liver disease and regulates proinflammatory cytokines expression.

### Effects of RA on NASH at the proteome level

**Fig. 4:**
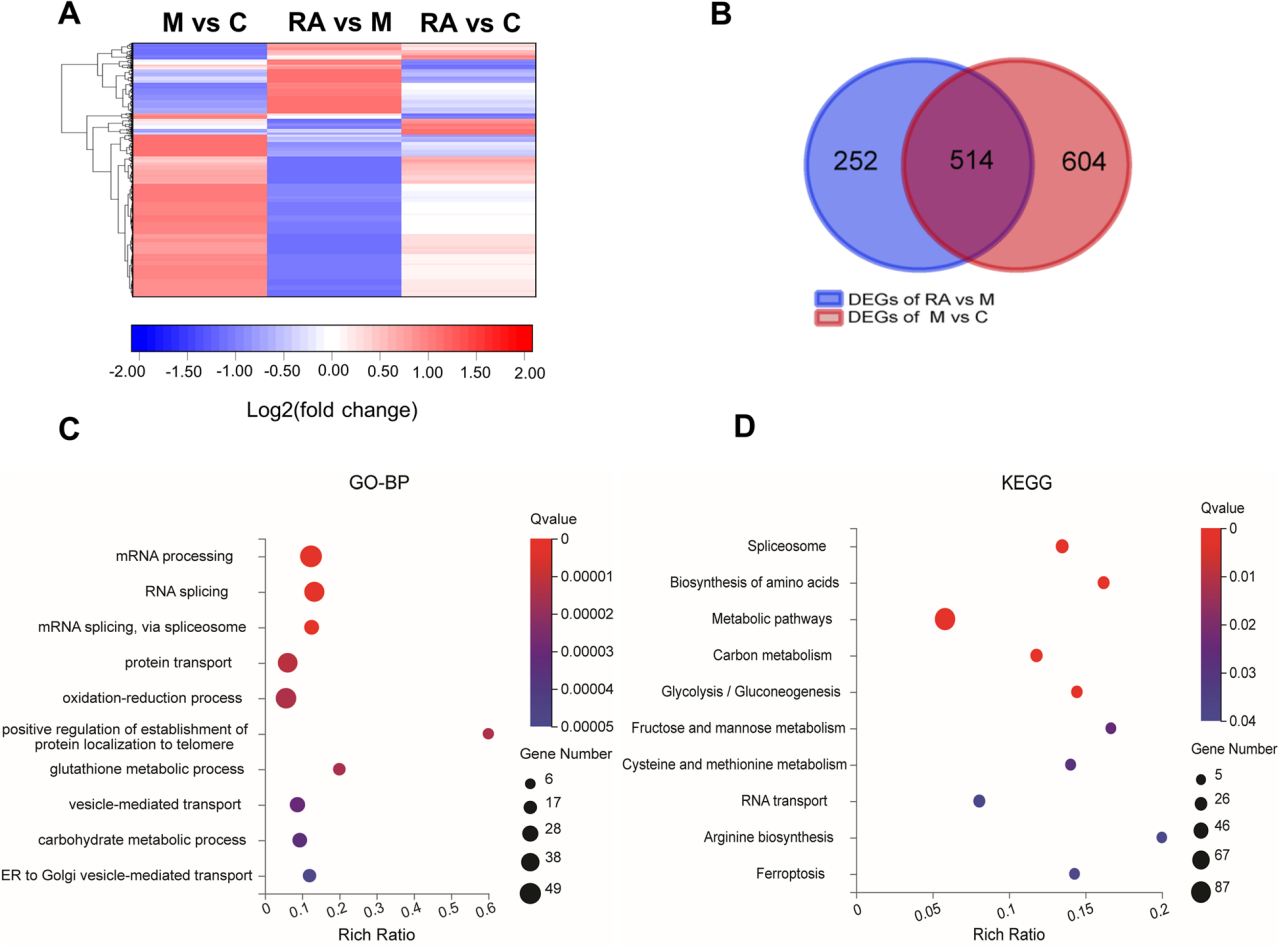
An overview of the proteomics of liver samples from HFD-fed mice and functional enrichment analysis of DEPs. **A** Heatmap displaying protein expression changes. Downregulated DEPs are shown in blue, and upregulated DEPs are shown in red. **B** Venn diagram of DEPs. **C-D** Top 10 categories for GO biological processes (**C**) and KEGG pathways (**D**) of DEGs depicted by bubble diagrams. The colour indicates the Q value, and the size indicates the gene number of each pathway. C: Control group. M: NASH group. RA: RA-30 group. *N* = 10 per group

In total, 15,958 peptides and 3493 proteins were identified for the DIA proteomic analysis (Supplementary Table 5). In total, 1118 proteins were differentially expressed in the NASH versus control comparison. Of these, 958 were upregulated and 160 were downregulated. In comparison with the NASH group, the RA group exhibited significant changes in 766 proteins (111 and 655 were upregulated and downregulated, respectively). Most proteins exhibited a smaller fold change in expression between the RA and control groups, indicating that the overall protein level tended to be normal after RA treatment (Fig. 4A). In total, 514 intersecting proteins were selected. (Fig. 4B and Supplementary Table 6).

The selected DEPs were assigned to GO categories to determine the biological processes in which they were involved. These DEPs were mainly associated with RNA processing and splicing, protein localization, amino acid metabolism, carbohydrate metabolism, and oxidation–reduction (Fig. 4C). The top 10 enriched pathways according to the KEGG enrichment study included carbohydrate metabolism, amino acid biosynthesis, RNA splicing and transport, and ferroptosis (Fig. 4D). The results of KEGG and GO analyses were consistent in terms of carbohydrate metabolism, amino acid metabolism, and RNA splicing. These pathways might be the key pathways for RA in hepatocytes.

### Network diagram analysis of proteomics

**Fig. 5:**
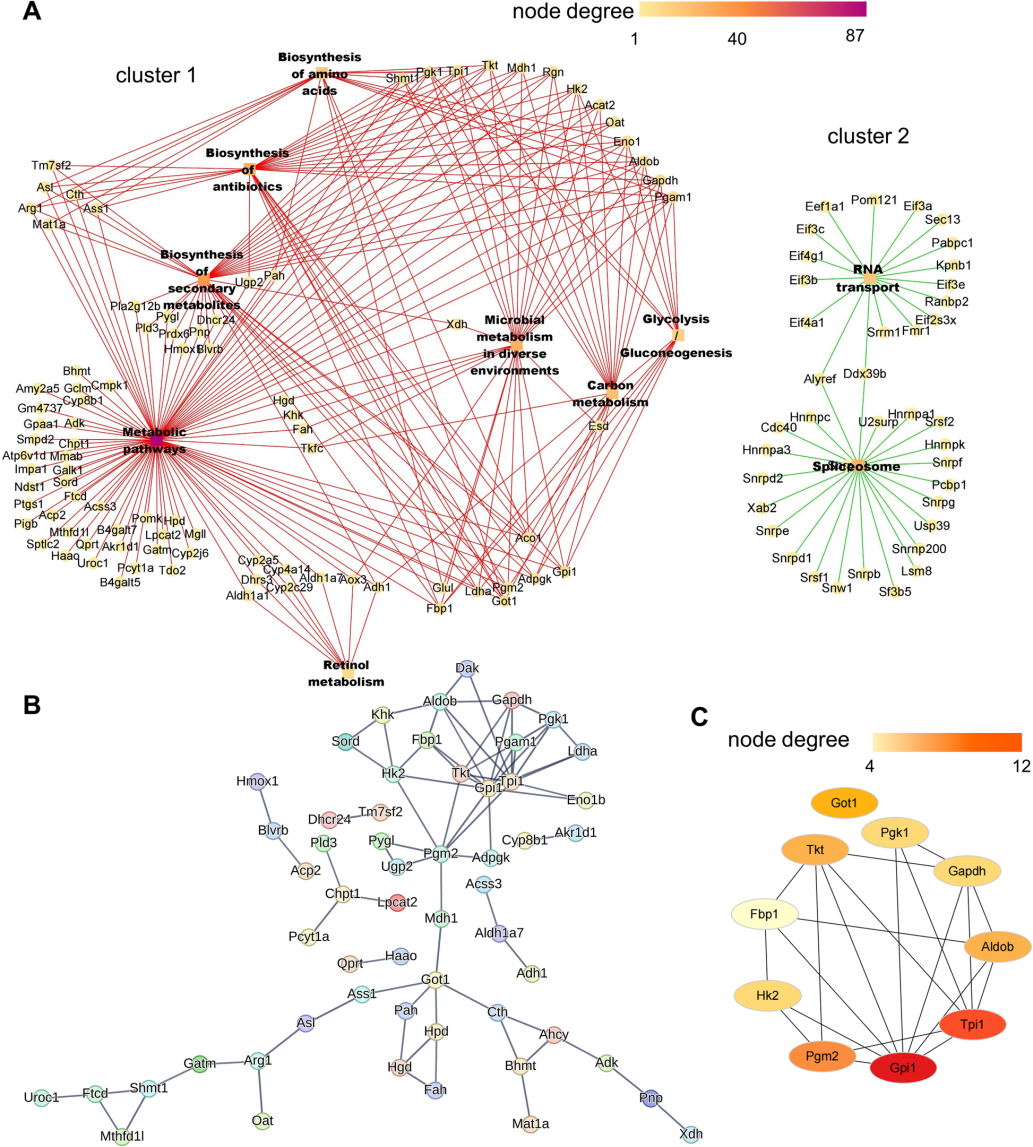
Top 10 hub genes of proteomics revealed by network analysis. **A** The pathway relation network of the top 10 KEGG pathways in DEPs. **B** The PPI network for DEPs in Cluster 1. **C** PPI hub genes ranked by degree in Cytoscape (ver.3.9.0). Node degree is represented by the redness of the nodes

Using the same method as previously, the top 10 pathways were selected to build the KEGG network based on the KEGG enrichment analysis. Two clusters were created from these paths (Fig. 5A). One cluster was mainly related to metabolic pathways and biosynthesis of amino acids and antibiotics, while the other cluster was related to RNA transport and the spliceosome.

Due to the fact that the complicated interaction of metabolic pathways in the liver is the basis of NASH pathogenesis, the metabolism-related module (Cluster 1) was selected for the protein interaction analysis. Protein interactions were analyzed using the STRING database. These interactions indicated that 88 DEPs were functionally linked with each other through 77 edges (Fig. 5B). Based on the degree in Cytoscape software, 10 hub proteins were selected. These proteins revealed the involvement of proteins associated with glucose metabolism: glucose-6-phosphate isomerase 1 (GPI1), triosephosphate isomerase 1 (TPI1), phosphoglucomutase 2 (PGM2), transketolase (TKT), aldolase B (ALDOB), HK2, glyceraldehyde-3-phosphate dehydrogenase (GAPDH), phosphoglycerate kinase 1 (PGK1), glutamic-oxaloacetic transaminase 1 (GOT1), and fructosebisphosphatase 1 (FBP1). These findings indicate that RA might strongly impact glucose metabolism (Fig. 5C and Supplementary Table 7).

### Integrative pathway based on proteome and transcriptome data analyses

**Fig. 6:**
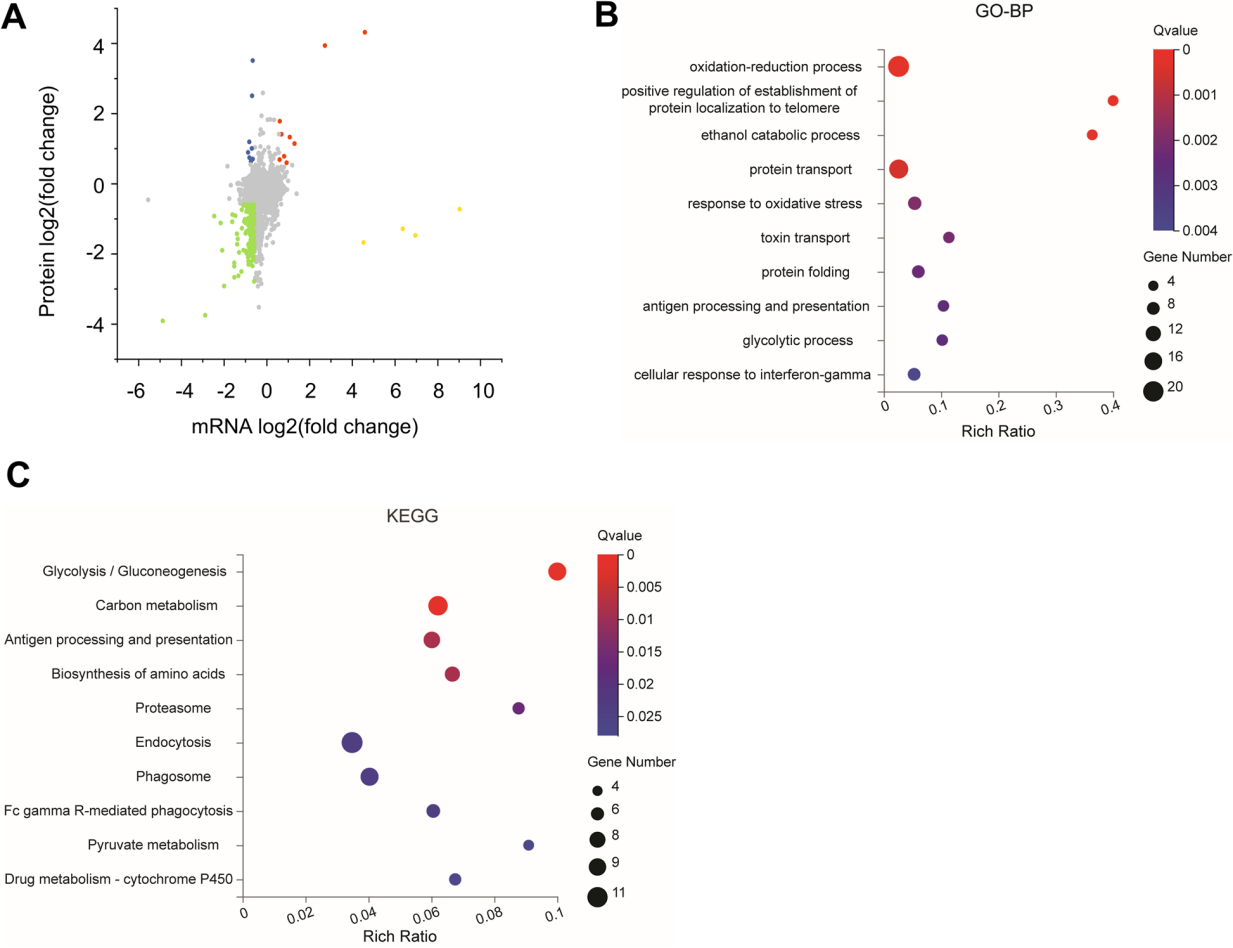
Integrative analysis based on proteome and transcriptome data. **A** Comparison of the expression changes in mRNA and protein. Blue: decreased mRNA and increased protein levels (*n* = 9); green: decreased mRNA and protein levels (*n* = 152); red: increased mRNA and protein levels (*n* = 9); yellow: increased mRNA and decreased protein levels (*n* = 4). **B-C** Top 10 GO biological process categories (**B**) and top 10 KEGG pathways (**C**) of DEPs/DEGs (green group), as depicted by bubble diagrams. The colour indicates the Q value, and the size indicates the gene number of each pathway. C: Control group. M: NASH group. RA: RA-30 group. *N* = 10 per group

To compare direction-related changes in mRNAs and proteins, 1576 proteins were identified that had corresponding mRNA data (FDR ≤ 0.001) in the NASH versus RA comparison, and their differences (fold change ≥ 1.5) were classified according to the direction of change (Fig. 6A and Supplementary Table 8). (Fig. 6A).

Functional enrichment analysis was applied to 154 genes concordant with decreasing mRNA and protein levels (green group). The GO analysis revealed that the most enriched processes were the oxidation–reduction process (response to oxidative stress), carbohydrate metabolism (ethanol catabolic process and glycolytic process), immune process (antigen processing and presentation, cellular response to interferon-γ), and protein transport and folding (Fig. 6B). Notably, according to the KEGG pathway analysis of the green group (*n* = 152) (Fig. 6C), glycolysis/gluconeogenesis was the first among the top 10 pathways ranked by the Q value. This finding was highly consistent with the proteome results showing that RA could affect glucose metabolism.

The expression patterns of the remaining groups (red, blue, and yellow) were visualized through a heatmap (Supplementary Fig. 4). Among these, cytochrome P450 CYP4A14 and CYP4A10 contribute to fatty acid oxidation [38–41] and their mRNA and protein levels were both greatly increased in the NASH group, consistent with previous studies [16]. This result indicated that RA could improve the fatty acid oxidation capacity in mice with NASH. Moreover, the level of insulin-like growth factor binding protein 2 (IGFBP2) is correlated with hepatic steatosis inversely [42], and both its genes and proteins were elevated in the RA group in the present study.

**Fig. 7:**
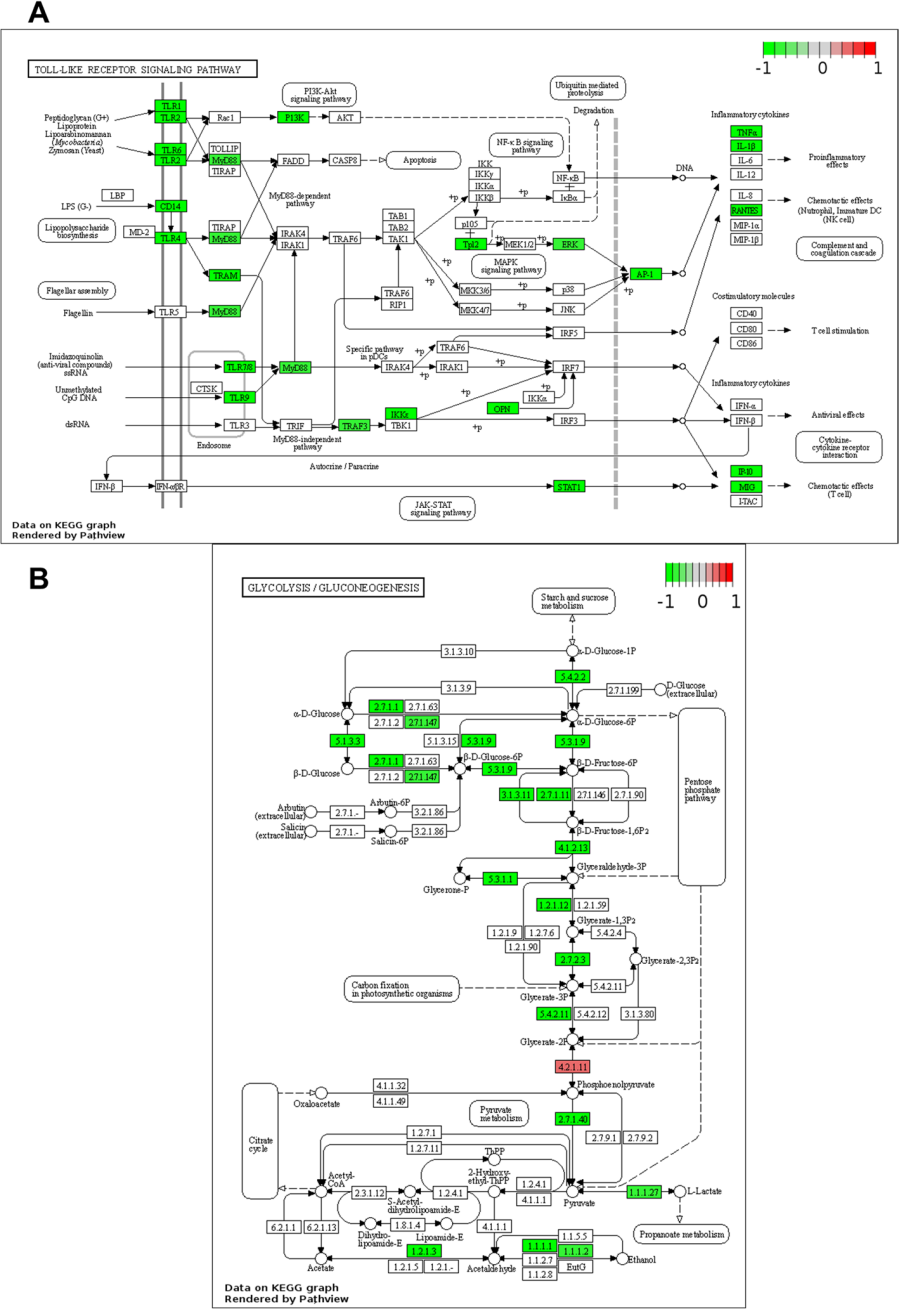
Integrated KEGG pathway maps. DEGs and DEPs were mapped to the Toll-like receptor signaling pathway (**A**) and glycolysis/gluconeogenesis (**B**) (the change in mRNA and protein expression is expressed as log2 [fold change])

Based on the protein and gene expression data, the GO and KEGG annotations and hub genes in the proteome and transcriptome analyses, and the correlation results, integrated pathway maps were constructed. Key genes in the TLR4/AP1 pathway and glycolysis were downregulated. After RA treatment, TLR4, MyD88, mitogen-activated protein kinase kinase kinase 8 (Map3k8, also named TPL2), mitogen-activated protein kinase 3 (Mapk3, also known as ERK), and AP1 subunits (c-Fos and c-Jun) were significantly downregulated (Fig. 7A and Supplementary Fig. 5). The key enzymes for glycolysis, that is, HK2, HK3, PFKL, PKM, and lactate dehydrogenase A (LDHA), were downregulated in the RA group versus the NASH group (Fig. 7B and Supplementary Fig. 6).

Therefore, the role of RA in improving NASH might be achieved through a decrease in glycolysis and the TLR4/AP1 pathway.

### Verification of the effect of RA on the TLR4/AP1 pathway

**Fig. 8:**
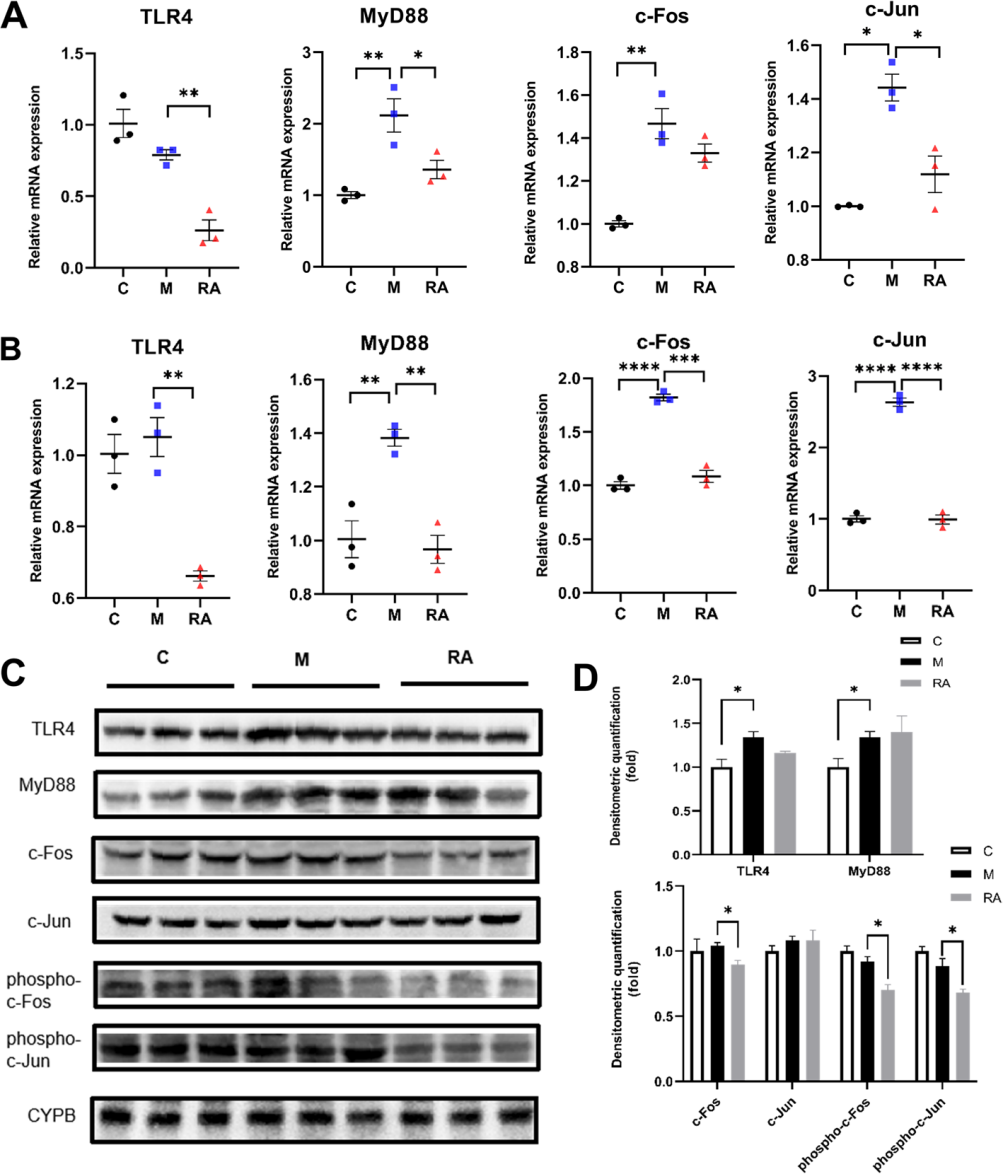
Effect of RA on the TLR4/AP1 pathway. **A-B** mRNA expression of TLR4, MyD88, c-Jun, and c-Fos in primary hepatocytes (**A**) and in primary Kupffer cells (**B**). **C-D** The protein abundances (**C**) and protein expression (**D**) of TLR4, MyD88, c-Fos, phospho-c-Fos, c-Jun, and phospho-c-Jun in primary Kupffer cells. *P < 0.05, **P < 0.01, ***P < 0.001, ****P < 0.0001 *vs.* the NASH group. C: Control group. M: NASH group. RA: RA-30 group. *N* = 10 per group

Four key genes of the TLR4/AP1 signaling pathway were examined in primary hepatocytes: TLR4, MyD88, and AP1 subunits (c-Fos and c-Jun). The mRNA expression of MyD88, c-Fos, and c-Jun were markedly elevated in the NASH group relative to the control group (Fig. 8A). Downregulation of TLR4, MyD88, and AP1 subunits was observed in the RA group relative to the NASH group, which indicates that a crucial mechanism through which RA might suppress inflammation is by altering the mRNA expression of genes in the TLR4/AP1 signaling pathway in hepatocytes.

Most studies on TLR4 signaling have focused on nonparenchymal cells, such as Kupffer cells [43]. Therefore, the above-mentioned four key genes were examined in primary Kupffer cells.

In the HFD-induced NASH group, the relative MyD88, c-Fos, and c-Jun mRNA levels were markedly elevated by 38%, 82%, and 163%, respectively, compared with the control group. RA treatment reversed the increase in MyD88, c-Fos, and c-Jun mRNA levels and decreased TLR4 mRNA levels by 37% in comparison with those of the NASH group (Fig. 8B).

The RA-regulated TLR4/AP1 pathway was further explored by measuring the protein levels of TLR4, MyD88, c-Fos, phospho-c-Fos, c-Jun, and phospho-c-Jun. In comparison with the control group, a 34% increase was observed in the expression of TLR4 and MyD88 (*P* < 0.05), whereas a trend of increase in c-Jun and c-Fos expression was observed (Fig. 8C, D). After RA treatment, c-Fos, phospho-c-Jun, and phospho-c-Fos expression levels decreased by 14%, 23%, and 24% (*P* < 0.05), respectively, and TLR4 expression exhibited a decreasing trend. However, MyD88 and c-Jun expression remained unchanged. Because of the action of RA, TLR4 expression and AP1 phosphorylation decreased. Thus, RA could reduce inflammation in HFD-induced mice by modulating the TLR4/AP1 signaling pathway expression.

### Verification of the effect of RA on glycolysis

**Fig. 9:**
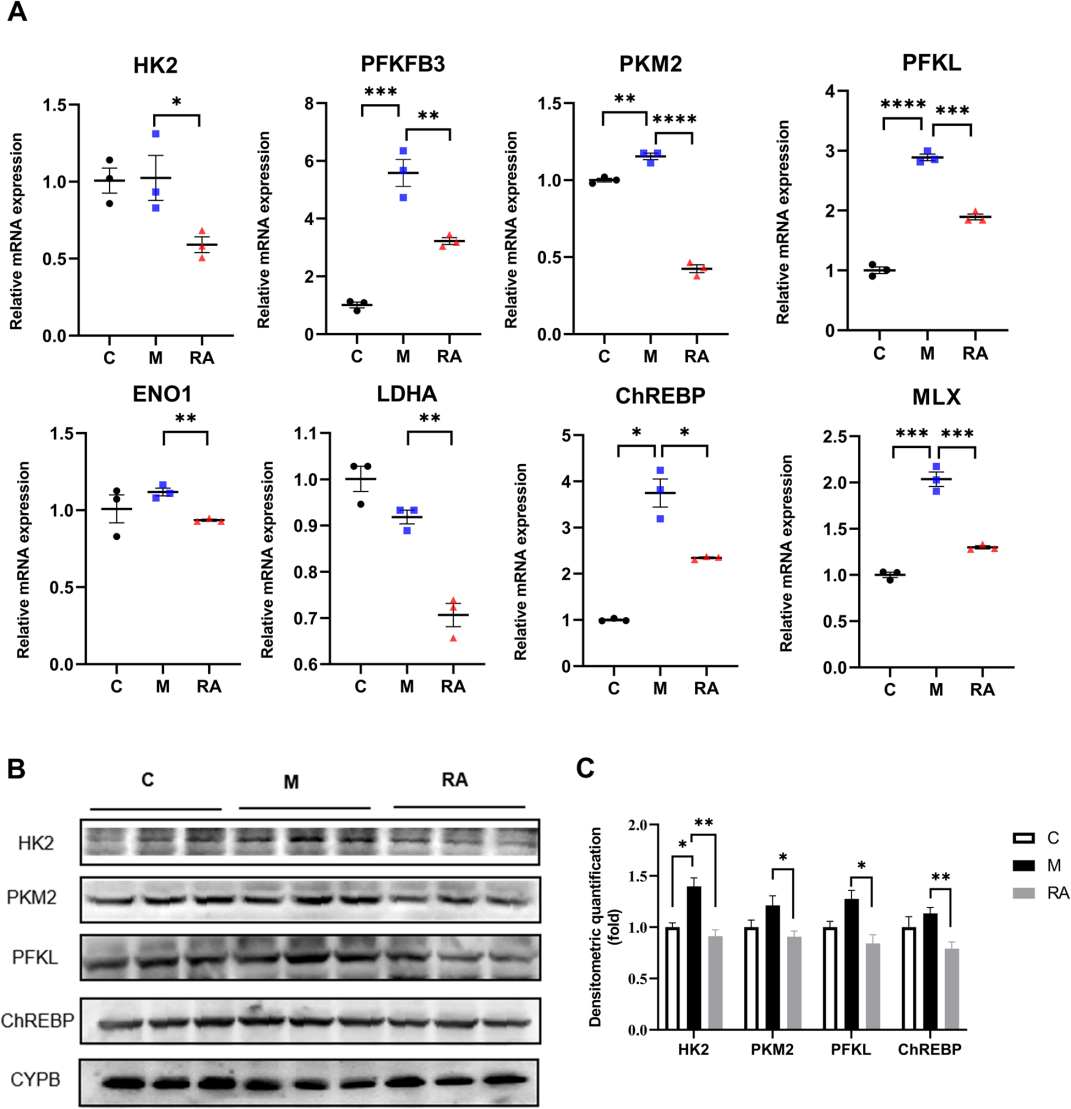
Effect of RA on the key factors involved in glycolysis. **A** The mRNA expression levels of hepatocyte HK2, PFKFB3, PKM2, PFKL, ENO1, LDHA, ChREBP, and MLX. **B-C** The protein abundances (**B**) and protein expression (**C**) of hepatocyte HK2, PKM2, PFKL, and ChREBP. *P < 0.05, **P < 0.01, ***P < 0.001, ****P < 0.0001 *vs.* the NASH group. C: Control group. M: NASH group. RA: RA-30 group. *N* = 10 per group

The DEGs/DEPs identified through transcriptomic and proteomic analyses revealed that key glycolytic enzymes were significantly regulated in the liver (Fig. 7B). The expression of glycolysis-related genes (HK2,6-phosphofructo-2-kinase (PFKFB3), PKM2, PFKL, and enolase 1 (ENO1)) was relatively quantified through qPCR. The RA group exhibited significantly decreased mRNA levels of the aforementioned genes in comparison with the NASH group, with the mRNA levels of PFKFB3, PKM2, and PFKL distinctly increased by 452%, 15%, and 188%, respectively, in the NASH group after the mice were fed a HFD (Fig. 9A).

LDHA is predisposed to converting pyruvate into lactate, which is the final step in glycolysis [44]. LDHA mRNA expression exhibited a decreasing trend in the NASH group relative to the control group and was substantially decreased in the RA group by 23% relative to the NASH group (*P* < 0.01). These data suggested that RA exerts a suppressive effect on glycolysis and changes LDHA mRNA expression (Fig. 9).

Glycolysis regulates the transcription factor ChREBP, which interacts with Max-like protein (MLX), thereby affecting the expression of lipid synthesis genes [45]. In this study, the ChREBP and MLX mRNA expression were significantly increased by 275% and 103%, respectively, in the mice fed a HFD. In contrast to the NASH group, RA treatment reversed the enhanced MLX and decreased ChREBP mRNA levels by 37% (Fig. 9A).

The aforementioned results were verified through western blotting to confirm HK2, PKM2, PFKL, and ChREBP expression levels in mouse primary liver cells. HK2 protein levels were enhanced by 40% after the mice were fed a HFD, whereas PFKL, PKM2, and ChREBP protein levels exhibited an upwards trend. Following RA treatment, HK2, PKM2, PFKL, and ChREBP expression decreased to the normal physiological level (Fig. 9B-C). Consequently, RA inhibited glycolysis and ChREBP to restore inflammation and lipogenesis in mice with NASH.

### Effect of RA on arsenic-induced glycolysis in vitro

To further confirm whether RA acts as an inhibitor of glycolysis, the effect of RA on arsenic-induced glycolysis in normal liver cells (L-02) was assessed according to published methods [46, 47].

**Fig. 10:**
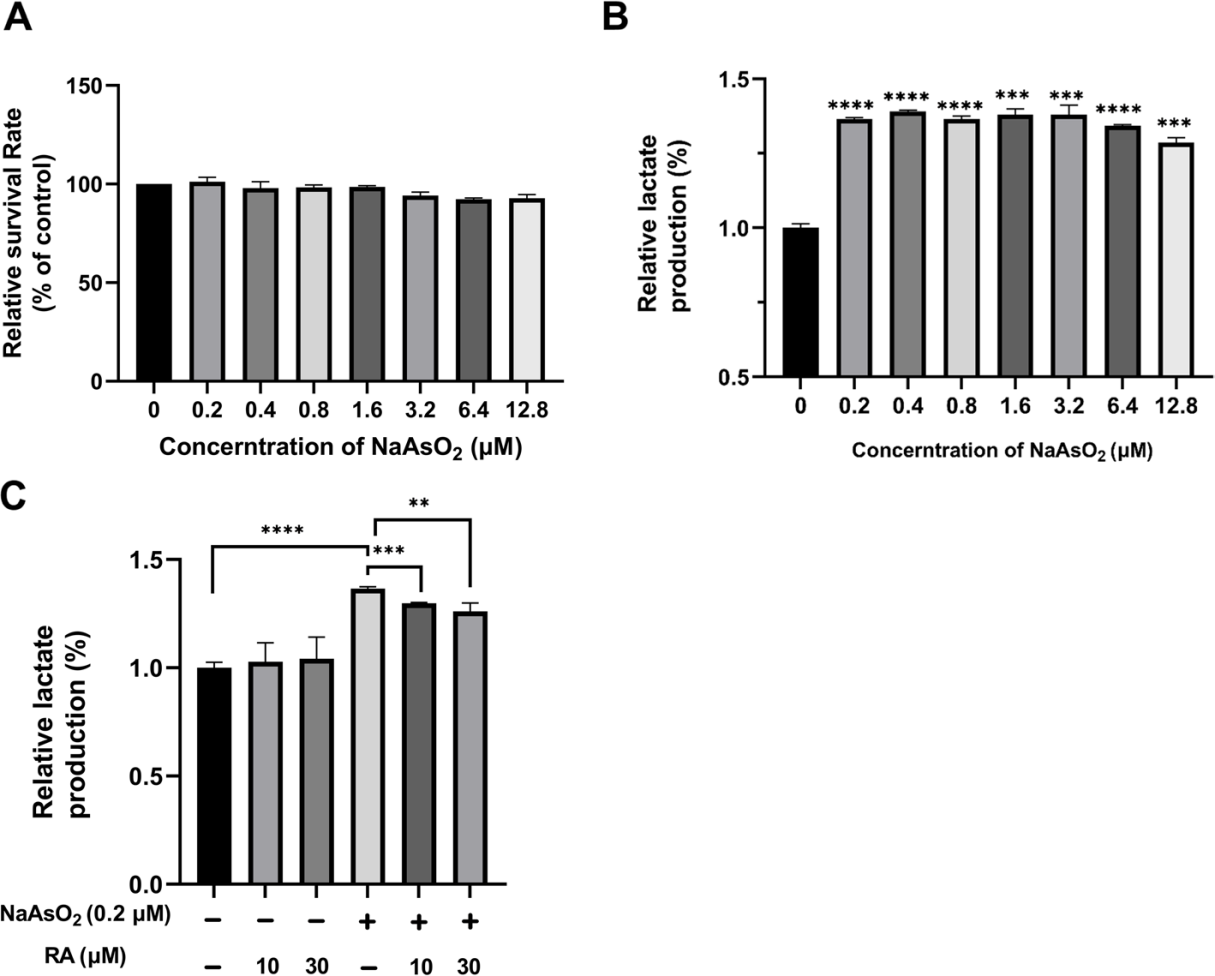
The effect of RA on glycolysis in NaAsO_2_-treated L-02 cells. **A** Viability of L-02 cells treated with various NaAsO_2_ doses for 24 h. **B** The release of lactate in L-02 cells treated with various NaAsO_2_ doses. *P < 0.05, **P < 0.01, ***P < 0.001, ****P < 0.0001 *vs.* 0 μM NaAsO_2_ treatment. **C** L-02 cells release lactate in response to NaAsO_2_ and RA. *P < 0.05, **P < 0.01, ***P < 0.001, ****P < 0.0001. *N* = 3 per group

NaAsO_2_ (0.2–12.8 μM) exhibited no conspicuous inhibitory effect on L-02 cell growth (Fig. 10A). Lactate production in these cells increased significantly after treatment with different NaAsO_2_ doses for 24 h (Fig. 10B).

The extracellular lactate level was enhanced in the NaAsO_2_-treated group relative to the control group (*P* < 0.001) (Fig. 10C). RA itself had no effect on lactate production. RA concentrations of 10 and 30 μM were used in the study according to a previous publication [16]. Compared with the NaAsO_2_-treated group, lactate production in the RA-treated group decreased dose-dependently (*P* < 0.01) (Fig. 10C). These studies revealed that RA reduces NaAsO_2_-induced lactate production in L-02 cells.

Overall, RA decreased glycolysis to reduce lactate production, thereby alleviating inflammation and excessive lipogenesis.

## Discussion

The study provided integrated links of expression patterns between the transcriptome and proteome in primary hepatocytes. These links uncover the detailed anti-NASH mechanism associated with glycolysis and TLR4/AP1 signaling pathways.

Inhibiting the TLR4/AP1 signaling pathway can attenuate hepatic inflammation and fibrosis in mice [48]. In the previous study, RA significantly suppressed inflammatory cytokines (IL-1β, IL-6, and TNFα) levels in HFD-fed mice [16]. Transcriptome analysis was conducted in this research to identify regulatory genes, which revealed that RA possibly suppresses the TLR4/AP1 signaling pathway.

Relative to the control group, the mRNA levels of TLR4, MyD88, c-Fos, and c-Jun were increased in the NASH group. Following RA treatment, the mRNA levels of these four genes were all decreased distinctly (*P* < 0.01), and the protein expression levels of c-Fos, phospho-c-Jun and phospho-c-Fos were significantly decreased (*P* < 0.05) (Fig. 8). Thus, RA decreased TLR4 expression and AP1 phosphorylation. TLR4 and MyD88 protein expression did not significantly decrease in the RA group in comparison with the NASH group. These results could be explained on the basis of a previous study that showed that RA can block TLR4 dimerization and exert anti-inflammatory effects on pulmonary inflammation [18].

Therefore, RA can be reasonably concluded to reduce TLR4 expression and activation, thereby decreasing the phosphorylation of its downstream molecules, exhibiting an inhibitory effect on AP1 translocation, reducing inflammation, and alleviating NASH.

ChREBP, a key factor in glucose-mediated regulation of lipogenic genes expression, plays a vital part in lipid synthesis. This molecule interacts with MLX and regulates the expression of lipid synthesis genes [49]. This study demonstrated that ChREBP mRNA and protein levels both decreased in the RA group relative to the NASH group (Fig. 9). Reducing ChREBP expression decreases the expression of ACC1 and FAS, which are downstream genes [16]. These genes are linked to NASH development and play a crucial role in fatty acid synthesis.

Furthermore, ChREBP strongly regulates glycolytic and lipogenic pathways [50]. Glycolytic metabolites can activate ChREBP and cause its nuclear translocation. Several rate-limiting enzymes, such as HK, PFK, and PKM, dominate the glycolysis rate. Inhibiting glycolysis can reduce hepatic steatosis, inflammation, and fibrosis [51, 52]. The results revealed an increase in both mRNA and protein levels of HK2, PFKM, and PKM in the NASH group. Conversely, there was a noticeable downregulation of these molecules in the RA group in comparison with the NASH group (Fig. 9).

Therefore, RA might inhibit ChREBP through glycolysis and regulate genes in relation to fatty acid synthesis.

Lactate is one of the pyruvate products formed during glycolysis, and LDHA is predisposed to converting pyruvate into lactate [44]. Lactate causes inflammation and lipid accumulation [53, 54]. In this study, RA markedly suppressed LDHA expression in vivo (Fig. 9A) and inhibited lactate release, which was increased with NaAsO_2_ treatment in vitro (Fig. 10). Thus, the effects of RA on lactate production were attributable to the downregulation of LDHA expression and glycolysis in hepatocytes, which then reduced inflammation and lipid accumulation in mice.

In the liver, ChREBP and SREBP-1c cooperate to induce fatty acid synthesis [55]. According to the early study results [16, 56], RA could attenuate triglyceride synthesis through inhibiting the SREBP-1c/SCD1 pathway and activating the AKT/mTOR pathway, which has been implicated in SREBP activation. The AKT/mTOR pathway promotes the accumulation of the active form of SREBP1 [57, 58]. Additionally, AKT/mTOR increased the glycolysis rate [59, 60].

**Fig. 11:**
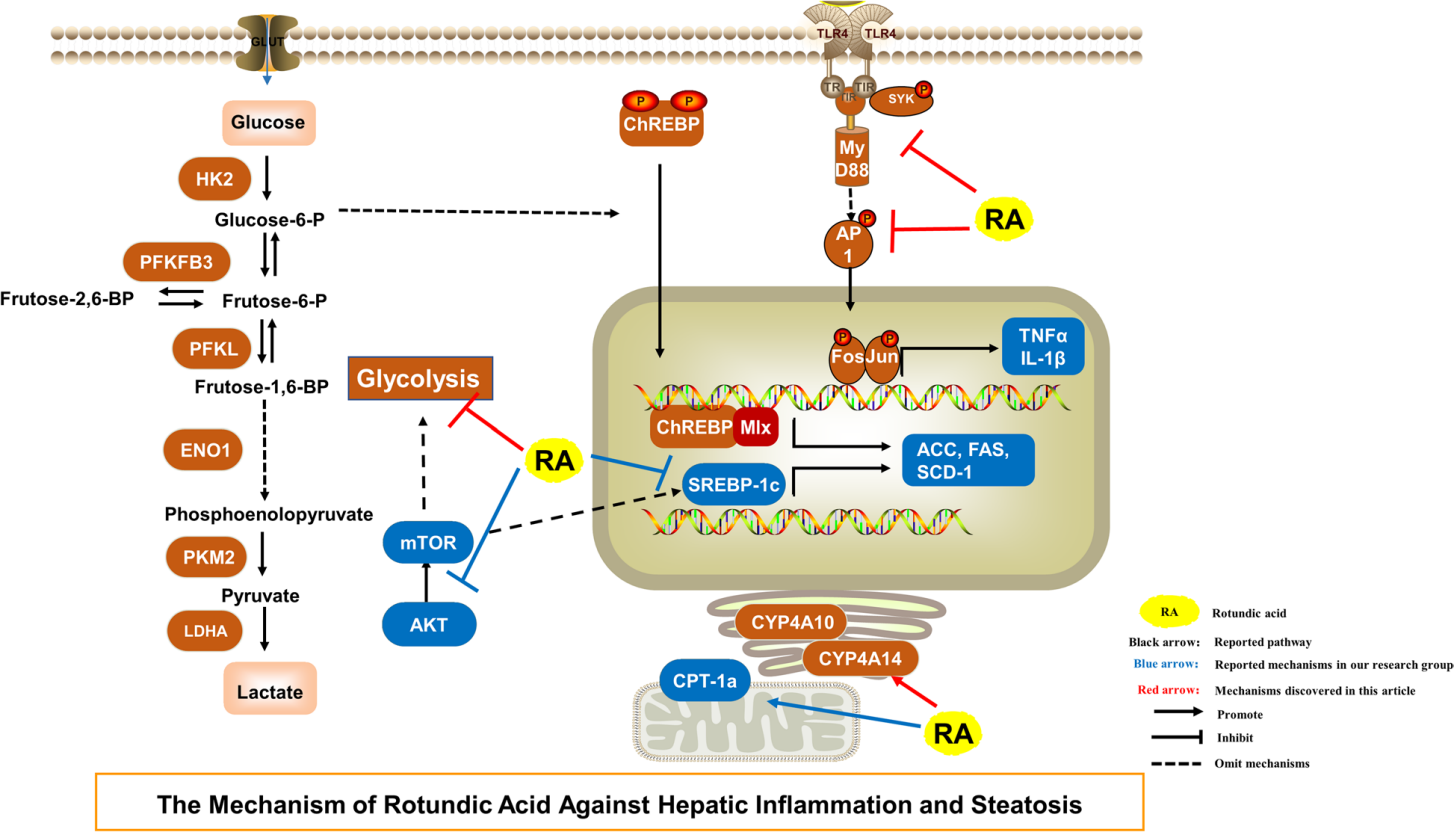
The proposed mechanism of action of RA on NASH. Black arrows represent the reported pathways, including the TLR4/AP1 signaling pathway, SREPB1c/SCD1 signaling pathway crosstalk with ChREBP, glycolysis metabolism, and the AKT/mTOR pathway. Blue arrows represent the reported mechanisms of RA. Red arrows represent the findings of this study

In summary, RA regulates inflammation and fatty acid synthesis through multiple pathways. The TLR4/AP1 and glycolysis pathways are among the most crucial regulatory pathways, along with the currently known mechanism of action (Fig. 11).

### Comparisons with other studies and what does the current work add to the existing knowledge

RA efficiently treats metabolic diseases including hyperglycaemia, hyperlipidaemia, diabetes, and cardiovascular disease [16, 61, 62]. However, the possible mechanisms of action of RA need to be further investigated to support RA development. Using in vivo and in vitro models, this study systematically indicated that RA improves NASH by regulating glycolysis and the TLR4/AP1 signaling pathways. Furthermore, inhibiting glycolysis can reduce hepatic steatosis, inflammation, and fibrosis [52, 53]. However, glycolysis-targeting pharmacological agents for NASH treatment are lacking [4]. The study findings presented a potential therapeutic reagent (RA) and an attractive novel therapeutic target (glycolysis) for NASH.

### Study strengths and limitations

This study integrates preliminary research and establishes a map of the mechanism of action of RA on NASH. Primary liver cells, as the most crucial parenchymal cell type in the liver, was selected for transcriptomic and proteomic studies. This method can more economically and conveniently elucidate the effects of drugs on the liver and avoid the interference of other factors. This study had several unresolved issues that require further investigation. First, whether RA-induced alterations had a direct effect on nuclear receptors or indirect regulation through relevant adaptor proteins in addition to TLR4 in the TLR4/AP1 pathway remains unclear. Second, the present study analyzed the alteration of glycolysis, but how RA regulates the glycolytic pathway remains unelucidated. Future related studies should focus on addressing these issues.

## Conclusions

These findings systematically demonstrate that RA targets signaling pathways, specifically ameliorating glycolysis and TLR4/AP1 pathways in NASH. This study broadens the strategies for treating NASH through the manipulation of glucose metabolism and metabolic inflammation and offers new ideas for analyzing anti-NASH mechanisms of RA at the molecular level, which will likely stimulate further advancements in the pharmaceutical industry.

### Supplementary Information

**Additional file 1.**

**Additional file  2.**

**Additional file 3.**

**Additional file 4.**

**Additional file 5.**

**Additional file 6.**

**Additional file 7.**

**Additional file 8.**

**Additional file 9.**

**Additional file 10.**

**Additional file 11.**

## Data Availability

All data generated or analyzed in this study are available from the corresponding author for the reasonable request.

## Abbreviations

NASH: Nonalcoholic steatohepatitis
NAFLD: Nonalcoholic fatty liver disease
RA: Rotundic acid
HFD: High fat diet
DDA: Data dependent acquisition
DIA: Data independent acquisition
GO: Gene Ontology
KEGG: Kyoto Encyclopedia of Genes and Genomes
DEGs: Differentially expressed genes
DEPs: Differentially expressed proteins
PPI: Protein − protein interaction
CYPB: Cyclophilin B
HK2: Hexokinase 2
PFKFB3: 6-Phosphofructo-2-kinase
ENO1: Enolase 1
PFKL: Phosphofructokinase
PKM: Pyruvate kinase
LDHA: Lactate dehydrogenase A
ChREBP: Carbohydrate-responsive element-binding protein
MLX: Max-like protein
TLR4: Toll-like receptor 4
MyD88: Myeloid differentiation primary response gene 88
AP1: Activating protein 1
TNFα: Tumor necrosis factor α
ACC1: Acetyl-coenzyme A carboxylase
FAS: Fatty acid synthase
SREBP1c: Sterol regulatory element-binding protein 1c
SCD1: Stearyl-coenzyme A desaturase 1
SRC: Rous sarcoma oncogene
RAC2: Rac family small GTPase 2
SYK: Spleen tyrosine kinase
PLCG2: Phospholipase C gamma 2
H2-Eb1: Histocompatibility 2
VCAM1: Vascular cell adhesion molecule 1
CXCL10: C-X-C motif chemokine ligand 10
GPI1: Glucose-6-phosphate isomerase 1
TPI1: Triosephosphate isomerase 1
PGM2: Phosphoglucomutase 2
TKT: Transketolase
ALDOB: Aldolase B
GAPDH: Glyceraldehyde-3-phosphate dehydrogenase
PGK1: Phosphoglycerate kinase 1
GOT1: Glutamic-oxaloacetic transaminase 1
FBP1: Fructosebisphosphatase 1
