# Supplementary material for: Rotundic acid improves nonalcoholic steatohepatitis in mice by regulating glycolysis and the TLR4/AP1 signaling pathway
Source: Lipids Health Dis. 2023 Dec 4;22:214. doi: 10.1186/s12944-023-01976-z (PMC10694891; doi:10.1186/s12944-023-01976-z)
Supplement: Supplementary file 1 — Additional file 1. [file 12944_2023_1976_MOESM1_ESM.doc]

**Supplementary materials 1**

**MATERIALS AND METHODS**

**Animal Experiments**

Animal experiments was prepared in our previous research. Seventy C57BL/6 male mice weighting 18–22 g (8 weeks old) were housed under standard pathogen-free conditions with a 12 h light/dark cycle and free access to food and water. Animals were allowed 1 week to acclimatize before experiments. The HFD consisting of 60% fat, 1.25% cholesterol and 0.5% cholate. Seventy mice were randomly distributed into seven groups (n = 10 per group) as follows: (1) Control group: mice were fed in a normal chow diet for 8 weeks and orally administered with vehicle (20 % propylene glycol and 80% deionized water) once per day from the fifth to the eighth week. (2) NASH group: mice were fed in high-fed-diet (HFD) that contained 60% fat, 1.25% cholesterol and 0.5% cholate for 8 weeks and exposed to vehicle as the previous group. (3) GS-0976 (positive control) group. (4) RA-10 group: mice were fed in HFD for 8 weeks and orally administered with RA (10 mg/kg/day) once per day from the fifth to the eighth week. (5) RA-30 group: mice were fed in HFD for 8 weeks and orally administered with RA (30 mg/kg/day) once per day from the fifth to the eighth week. (6) RA-100 group: mice were fed in HFD for 8 weeks and orally administered with RA (100 mg/kg/day) once per day from the fifth to the eighth week. (7) NC + RA group: mice were fed in normal chow for 8 weeks and orally administered with RA (100 mg/kg/day) once per day from the fifth to the eighth week.

**Preparation of Rotundic acid**

Firstly, the medicinal materials of Ilicis Rotundae Cortex were crushed and reflux extracted with ethanol. Then Ilicis Rotundae Cortex ethanol extract was filtered, concentrated and dried. Ilicis Rotundae Cortex ethanol extract, activated charcoal, and 95% ethanol solution were added to the three-neck flask. The mixture was stirred, refluxed, filtered and concentrated.

The 10.0g precipitation were dissolved in 20 mL methanol and 100 mL 2 mol/L sodium hydroxide solution, and the reflux reaction was carried out for 2 h. The reaction solution is then acidified with hydrochloric acid and left for filtration. Add 30 mL water and 10 mL ethanol to the filter slag, recrystallize twice at 50℃. The product was identified as Rotundic acid by NMR and mass spectrometry. High performance liquid chromatography was used to determine purity.
