# Supplementary material for: Rotundic acid improves nonalcoholic steatohepatitis in mice by regulating glycolysis and the TLR4/AP1 signaling pathway
Source: Lipids Health Dis. 2023 Dec 4;22:214. doi: 10.1186/s12944-023-01976-z (PMC10694891; doi:10.1186/s12944-023-01976-z)
Supplement: Supplementary file 2 — Additional file 2. [file 12944_2023_1976_MOESM2_ESM.doc]

**Supplementary figures**

**
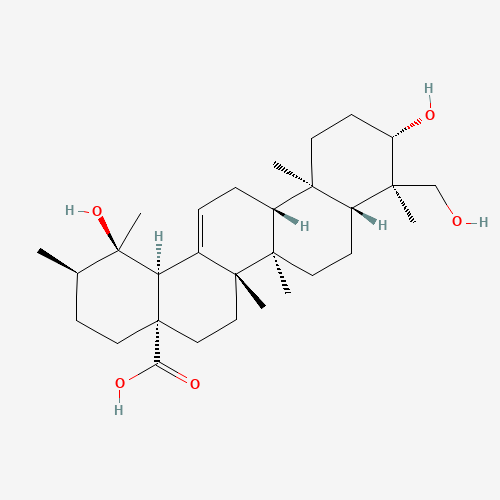
**

**Supplementary Fig. 1 Chemical structure of Rotundic acid.**


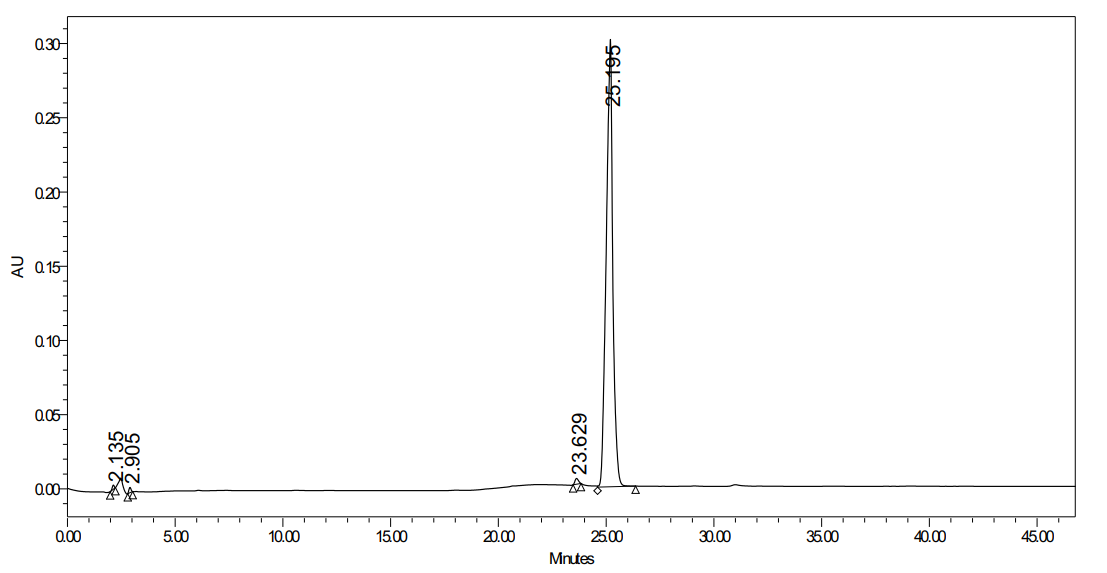


**Supplementary Fig. 2 The HPLC chromatogram of RA**


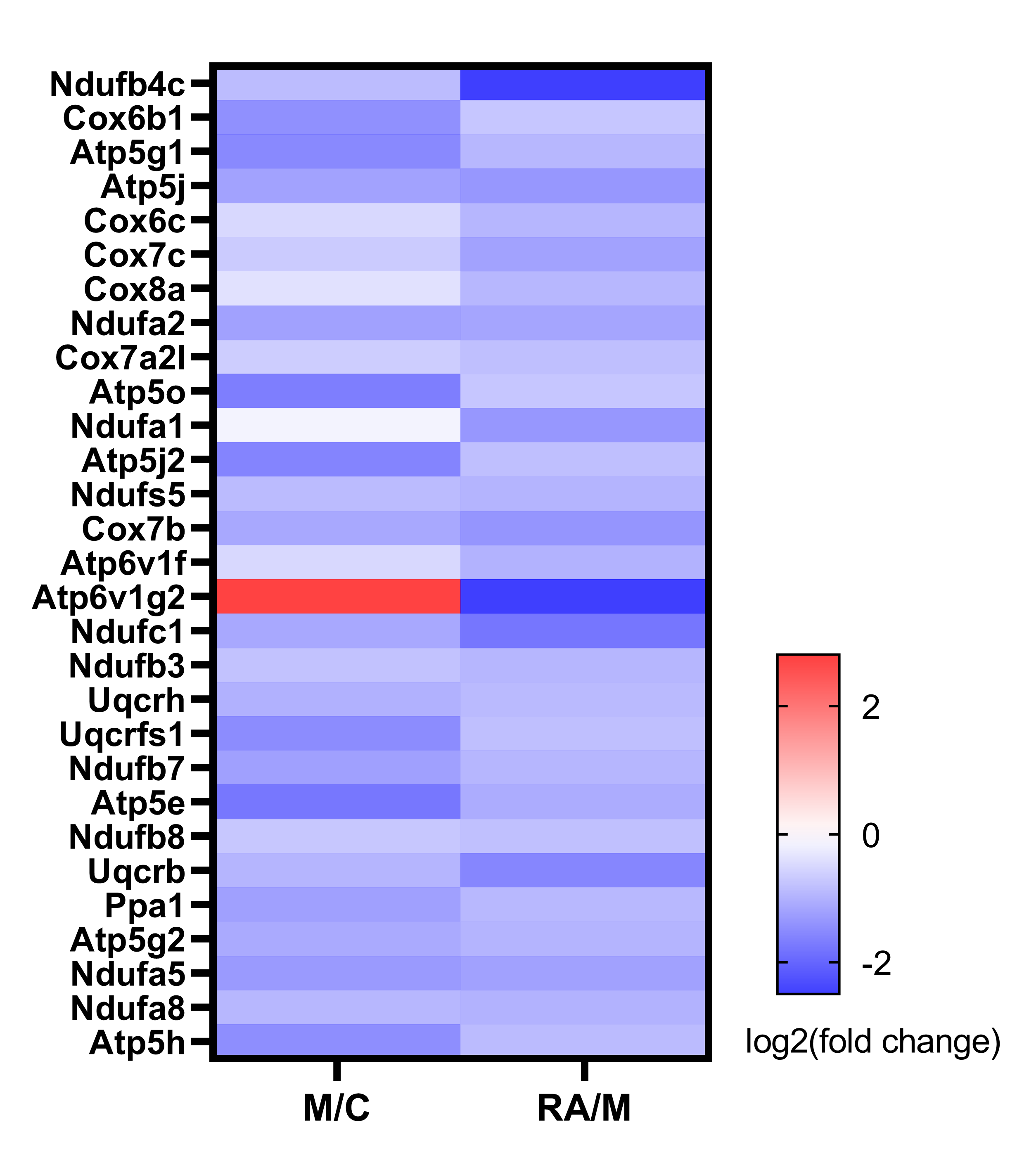


**Supplementary Fig. 3 Heatmap of expression of DEPs and DEGs in OXPHOS.**

**
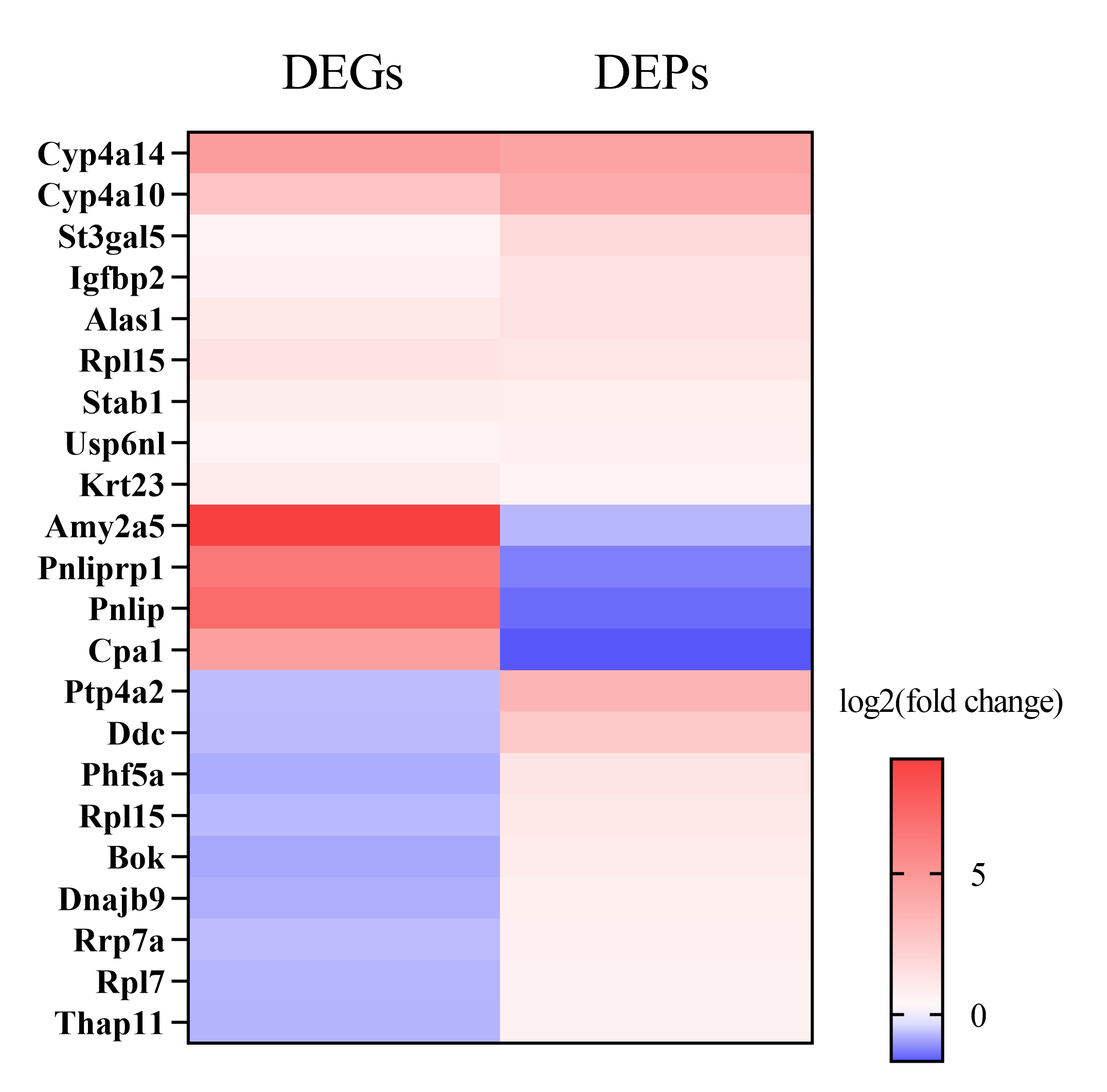
**

**Supplementary Fig. 4 Heatmap of expression of DEPs and DEGs in RA vs M (blue, red and yellow groups).**

**
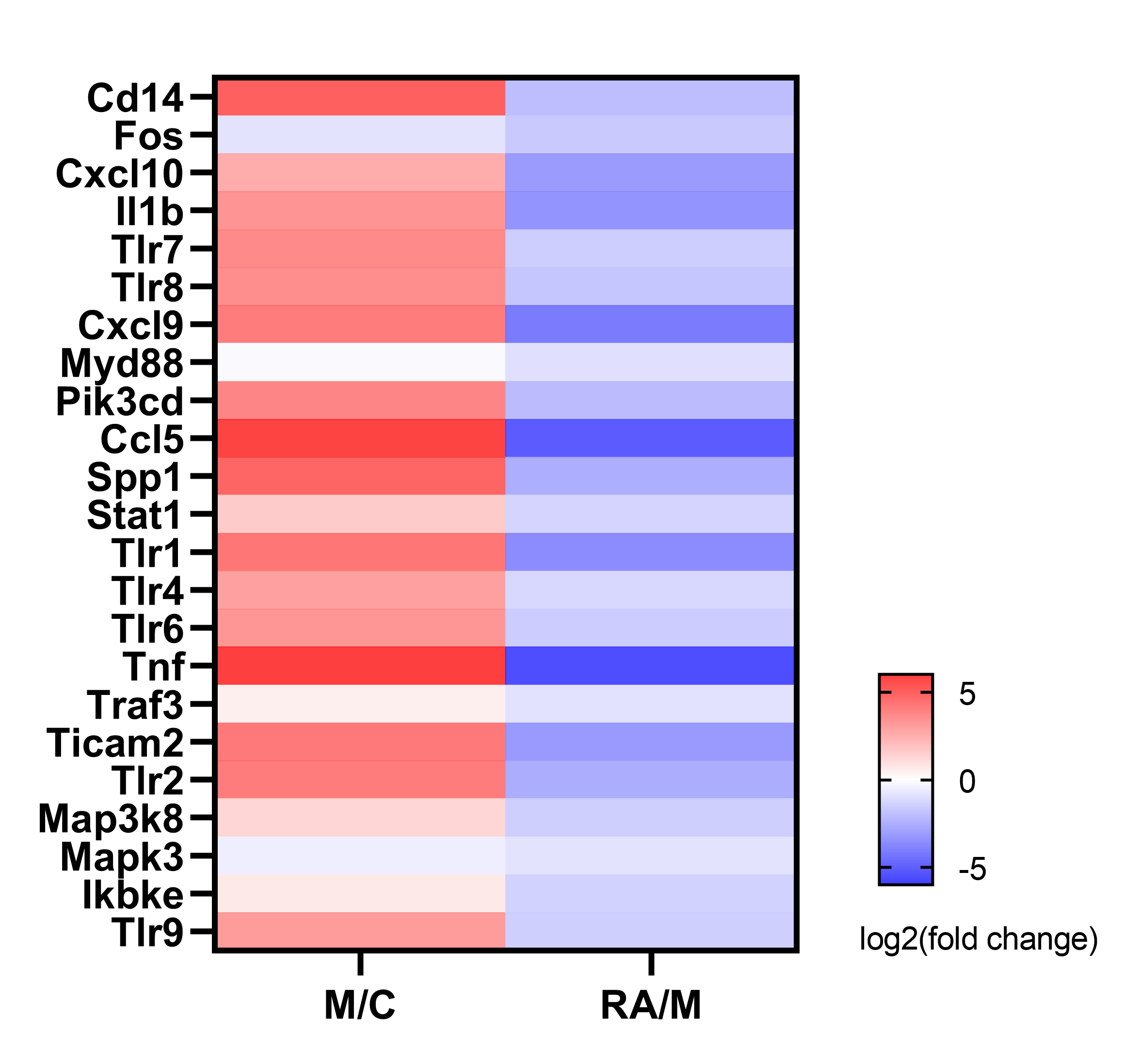
**

**Supplementary Fig. 5 Heatmap of the expression of the identified proteins/genes in Toll-like receptor signaling pathway.**

**
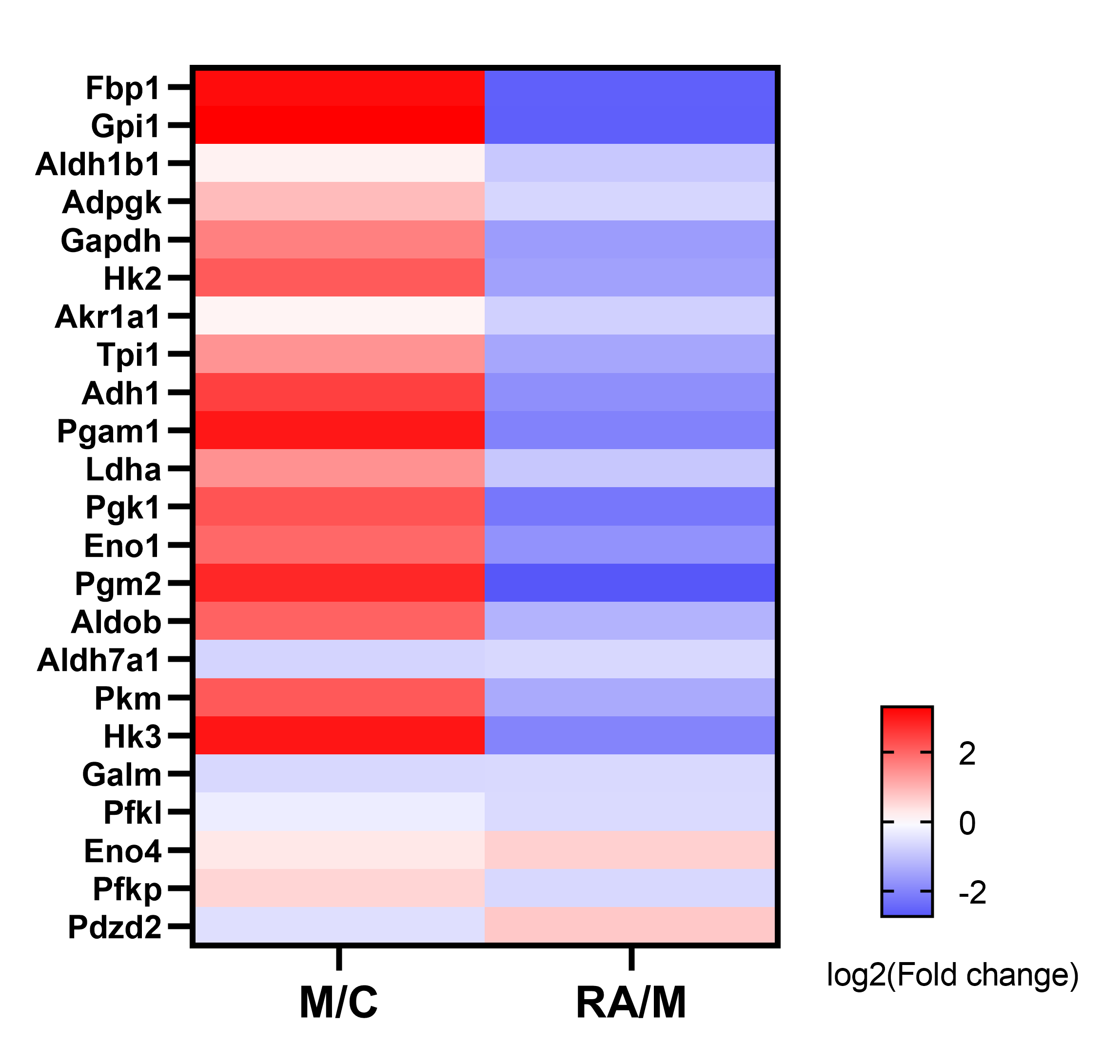
**

**Supplementary Fig. 6 Heatmap of the expression of the identified proteins/genes in** **glycolysis/gluconeogenesis.**
