# Supplementary material for: Rotundic acid improves nonalcoholic steatohepatitis in mice by regulating glycolysis and the TLR4/AP1 signaling pathway
Source: Lipids Health Dis. 2023 Dec 4;22:214. doi: 10.1186/s12944-023-01976-z (PMC10694891; doi:10.1186/s12944-023-01976-z)
Supplement: Supplementary file 11 — Additional file 11. [file 12944_2023_1976_MOESM11_ESM.doc]

**The original western blots images**


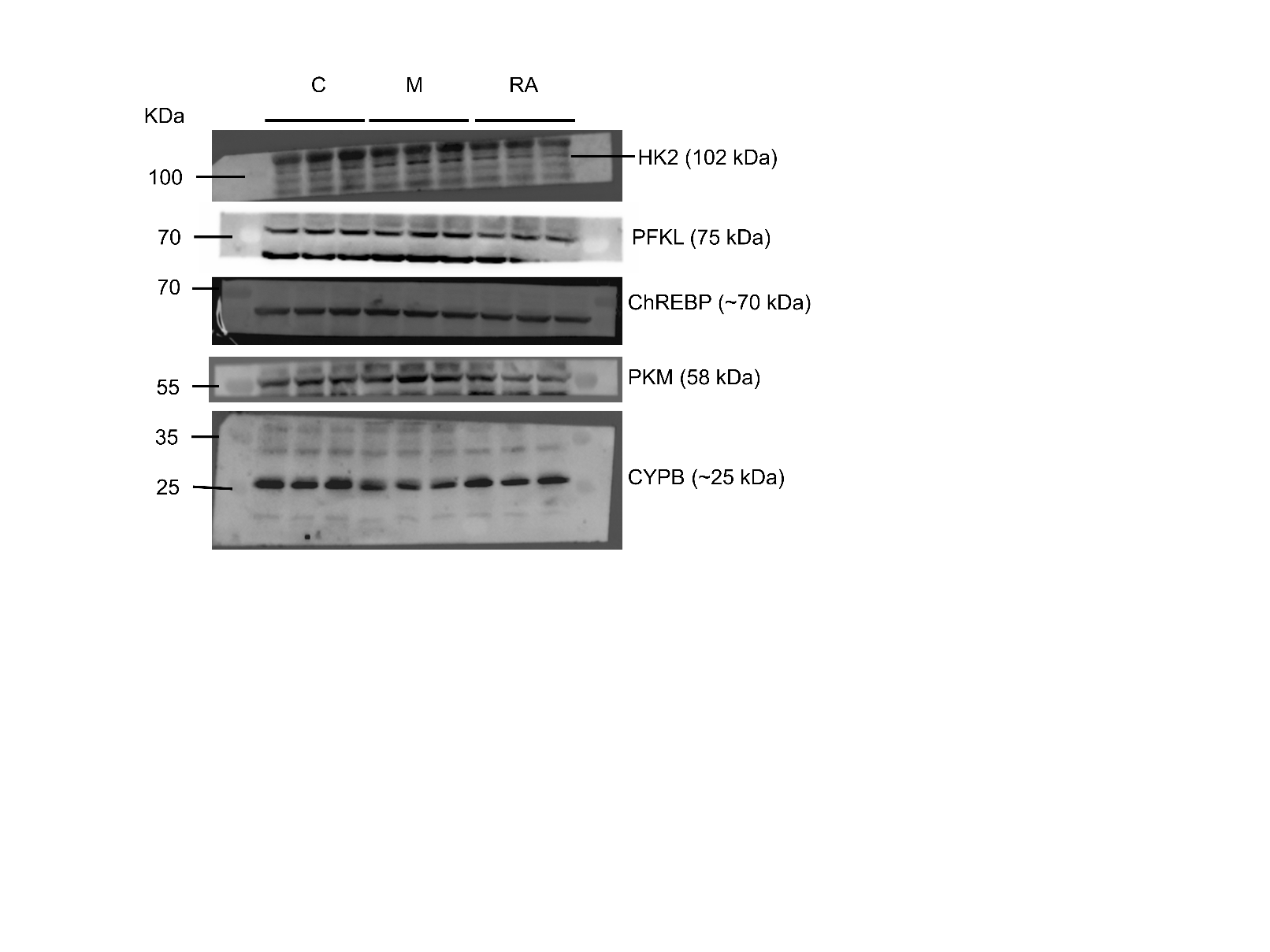


Protein abundances of hepatocyte HK2, PKM2, PFKL, ChREBP and CYPB.


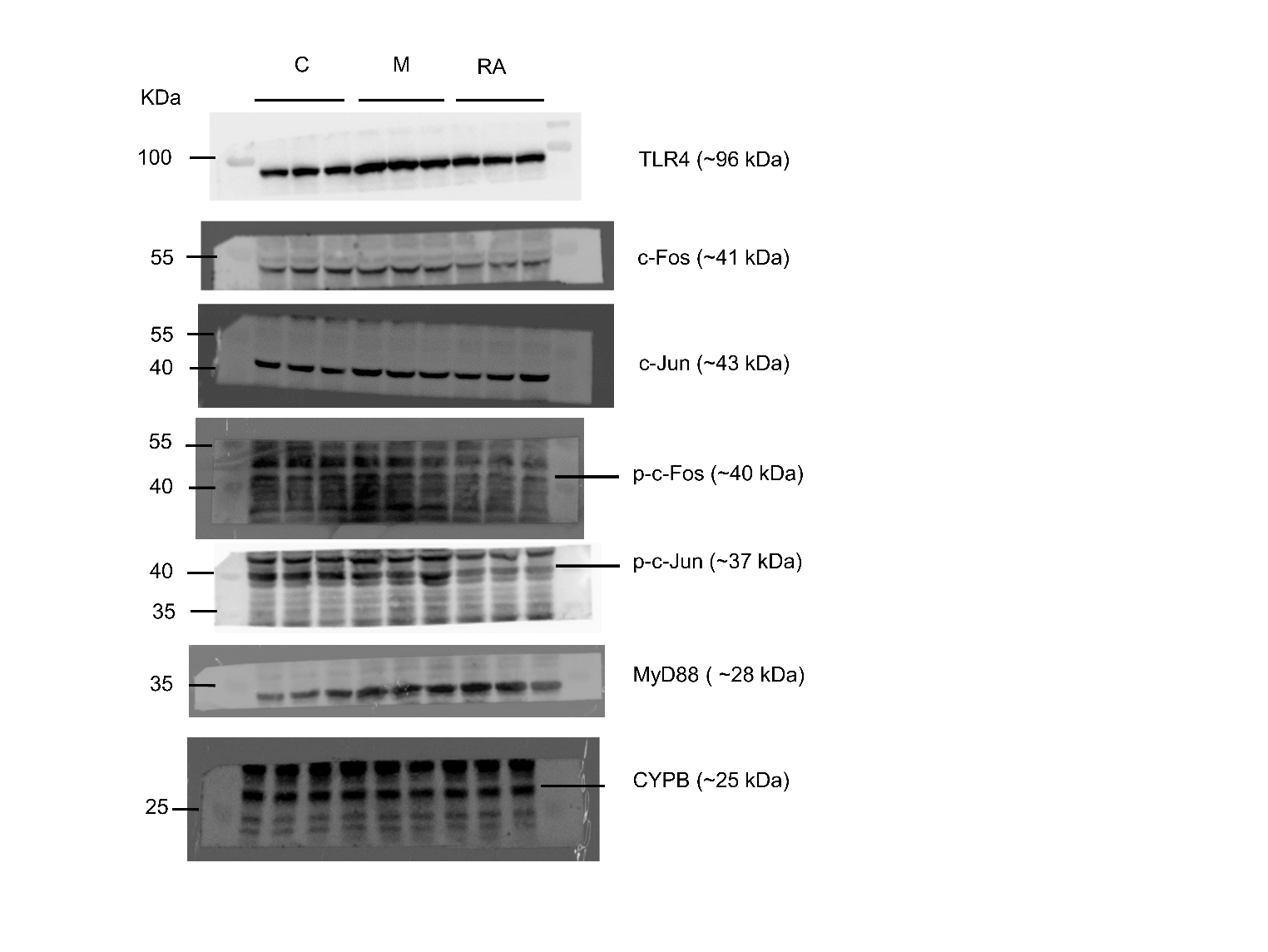


Protein abundances of TLR4, MyD88, c-Fos, c-Jun, phospho-c-Fos, phospho-c-Jun and CYPB
